# The effect of biofeedback interventions on pain, overall symptoms, quality of life and physiological parameters in patients with pelvic pain: A systematic review

**DOI:** 10.1007/s00508-021-01827-w

**Published:** 2021-03-22

**Authors:** Barbara Wagner, Margarete Steiner, Dominikus Franz Xaver Huber, Richard Crevenna

**Affiliations:** Department of Physical Medicine, Rehabilitation and Occupational Medicine, Medical University of Vienna, Waehringer Guertel 18–20, 1090 Vienna, Austria

**Keywords:** Pelvic floor dysfunction, Pelvic floor dyssynergia, EMG, Electromyography, Manometry

## Abstract

**Background:**

Biofeedback is recognized as an effective additive method for treating certain phenotypes of chronic pelvic pain syndrome and is a therapeutic option in other pelvic pain conditions. This review aims to evaluate evidence from the literature with a focus on the effect of biofeedback on pain reduction, overall symptom relief, physiological parameters and quality of life.

**Methods:**

A systematic literature search was conducted using the databases PubMed, MEDLINE, Embase, Cochrane Library and PEDro from inception to July 2020. Data were tabulated and a narrative synthesis was carried out, since data heterogeneity did not allow a meta-analysis. The PEDro scale and the McMaster Critical Review Form—Quantitative Studies were applied to assess risk of bias.

**Results:**

Out of 651 studies, 37 quantitative studies of primary research evaluating pelvic pain conditions in male and female adults and children were included. They covered biofeedback interventions on anorectal disorders, chronic prostatitis, female chronic pelvic pain conditions, urologic phenotypes in children and adults and a single study on low back pain. For anorectal disorders, several landmark studies demonstrate the efficacy of biofeedback. For other subtypes of chronic pelvic pain conditions there is tentative evidence that biofeedback-assisted training has a positive effect on pain reduction, overall symptoms relief and quality of life. Certain factors have been identified that might be relevant in improving treatment success.

**Conclusions:**

For certain indications, biofeedback has been confirmed to be an effective treatment. For other phenotypes, promising findings should be further investigated in robust and well-designed randomized controlled trials.

## Introduction

Biofeedback therapy is an instrument-based learning process employing operant conditioning. Autonomic and neuromuscular activity is measured and visual, acoustic and verbal feedback is provided to promote the acquisition of self-control over physiological processes, which are otherwise outside awareness or under less voluntary control [1].

Pelvic pain is perceived in pelvis-related structures and organs of either men or women and may be acute or chronic. In terms of chronic pelvic pain (CPP), there is no generally accepted definition. It can be subdivided into conditions with well-defined classical pathology and those with no obvious pathology—the chronic pelvic pain syndromes (CPPS). The European Association of Urology (EAU) describes CPPS as the occurrence of CPP with no proven infection or other obvious local pathology accounting for the pain, continuous or recurrent for at least 6 months. It is often associated with symptoms suggestive of lower urinary tract, sexual, bowel, gynecological or pelvic floor dysfunction and with negative cognitive, behavioral, sexual or emotional consequences [2].

Chronic pelvic pain is a common pain condition with a worldwide prevalence of 2.1–26.6% for noncyclic pain in women [3–5] and 2.2–9.7% in men [6].

Up to 85% of women with CPP have dysfunction of the musculoskeletal system, including spasm of the levator ani muscle [7]. Myofascial pelvic pain is a major component of CPP which is not always properly identified by healthcare providers [8]. It may be a primary or contributing source of CPP [8]. Its hallmark diagnostic indicators are myofascial trigger points in the pelvic floor musculature that refer pain to adjacent sites [8]. They are thought to occur in response to acute and chronic physical or psychosocial stress or trauma [9].

The pathophysiology of CPP is not well understood. Treatment is therefore often unsatisfactory and limited to symptom relief [7]. Several nonsurgical strategies exist that include medical, psychological, cognitive, behavioral, complementary and physical therapy [5, 7, 10]. In the case of myofascial pelvic pain in particular, a multidisciplinary team of specialists [8] and a multimodal treatment strategy are warranted. In a large proportion of patients, treatment does not necessarily result in pain relief. CPP therefore carries a significant physical, mental, and social burden for patients and puts a heavy burden on healthcare systems worldwide. Increased medical attention to identify and test effective treatment strategies is warranted [5, 7, 10, 11].

Biofeedback seems to be a promising adjuvant tool in the cognitive-behavioral treatment of somatoform disorders because it aims to enhance control over the psychophysiological processes that may be involved in these conditions [1]. Biofeedback is also one of several effective physical therapy techniques used to treat myofascial pelvic pain [8]. The recent EAU guidelines 2019 on CPP state that biofeedback is the preferred treatment for chronic anal pain and can improve the outcome of myofascial therapy as an adjuvant to muscle exercises in patients with hypertonic pelvic floor dysfunction [2]. It is considered a treatment option in type III chronic prostatitis according to the National Institutes of Health (NIH) classification [10].

Previous systematic reviews have evaluated the evidence of physiotherapy interventions in general in the management of CPP [5, 12, 13]. One review focused on the effect of biofeedback on improving symptoms of pelvic floor dysfunction in 2008 [14]. The primary aim of our review was to evaluate the effect of biofeedback interventions on subjective outcome pain, overall symptom improvement and quality of life in patients with acute or chronic pelvic pain conditions. A secondary aim was to investigate whether biofeedback interventions improved physiological parameters indicative of pelvic floor muscle tone and/or general relaxation.

## Methods

### Protocol and registration

A systematic review of the existing scientific literature was conducted, based on the guidelines recommended by the Preferred Reporting Items for Systematic Reviews and Meta-Analysis (PRISMA) statement [15]. The review protocol was registered (PROSPERO registration number: CRD42020201751).

### Identification and selection of studies

The search included the electronic databases PubMed, Medline, Embase, PEDro and Cochrane Library. Trials with the keywords “pelvic pain AND biofeedback” were extracted and considered for inclusion. No filters were used. No restrictions were placed on the year of publication. A systematic literature search was independently performed by two researchers (BW, MS) and disagreements in selection were resolved through discussion. The process was supervised by an experienced senior researcher (RC).

### Inclusion and exclusion criteria

Table 1 presents the inclusion and exclusion criteria regarding study design, participants, interventions and outcome evaluation. Any quantitative study type of primary research (with the exception of case studies/case series less than 10 participants) was included to present a comprehensive overview of the current literature. This approach goes along with previous reviews [5, 13] which stated that including randomized controlled trials (RCTs) only was not feasible in reviewing physiotherapy interventions in patients with CPP. We considered males and females of all ages with either acute or chronic pelvic pain conditions as listed in the EAU guidelines [2], including both specific disease-associated pelvic pain and pelvic pain syndromes. Interventions were judged eligible if biofeedback was administered as a sole intervention or significant component of a multimodal or multidisciplinary intervention (including mechanical or electrical devices), as multidisciplinary management of CPP is considered optimal [13].

**Table 1:** Inclusion and exclusion criteria

	Inclusion criteria	Exclusion criteria
Study design, comparison	Any quantitative study type of primary research with exception of case studies/case series < 10 participants; retrospective studies are included	Reviews, cross-sectional studies, case reports/case series < 10 participants, conference papers and abstracts, book chapters, editorials
	Control interventions may include treatment as usual, no treatment, surgery, medicinal treatment, physical therapy modalities or placebo treatment	Unconcluded studies, studies with missing outcome data
		Studies that were published in languages other than English and German
Participants	People with chronic pelvic pain according to the guidelines on chronic pelvic pain [2] including constipation (conditions with overactive pelvic floor dysfunction)	Pelvic organ prolapse, fecal or urinary incontinence (hypotonic pelvic floor dysfunction)
	People with acute pelvic pain	
	Males and females	
	Children, adolescents, adults	
Interventions	Biofeedback as a sole intervention or as a significant component of a multimodal intervention	No BFB-assisted training performed/BFB was not a relevant component of the treatment
	Clinical (in/outpatient) setting or home-based training	If only a subgroup of the study population received BFB: studies were excluded if the number of subjects in the BFB subgroup or the outcome results of this subgroup were not stated
		Insufficiently documented BFB intervention: no information on the training extent (frequency, number of sessions or duration of a single session) or the mode of application
Outcome measures	Primary outcome: – Pain intensity – Overall symptom improvement – Quality of life	–
	Secondary outcome: – Physiological parameters, indicative of pelvic floor muscle tone or general relaxation	

*BFB* biofeedback

### Data collection and analysis

For eligible papers, the following data were extracted: study characteristics (author and year of publication, country site, study design, sample sizes, drop-out rate, diagnoses investigated, author’s conclusions), patient characteristics (sample characteristics, mean duration of symptoms, sex, mean age), intervention characteristics (interventions administered, time points of follow-up, biofeedback devices, training extent, intervention setting, adverse events) (Tables 2, 3 and 4). Primary outcome parameters were pain, overall symptom improvement and quality of life. Secondarily, physiological parameters were assessed. When certain data were not given in the respective studies the information was stated as “not available”.

**Table 2:** Study characteristics

Study	Country	Study design (details: see Table 8)	*n* of IG receiving BFB (*n* of whole study participants)	Drop-outs at last f/u/excluded from analysis	Diagnose(s), symptoms:	Conclusions by authors of respective papers
**Anorectal pain syndrome**						
Chiarioni et al. 2010 [16]	Italy	RCT (3 arm): 3 different interventions compared	52 (157)	n.a.	Levator ani syndrome (constipation excluded)	BFB is superior to EGS and levator ani massage in pain relief. Improvements maintained for 12 months. Only patients with tenderness on rectal examination benefit. Pathophysiology of levator ani syndrome pathophysiology is similar to dyssynergic defecation type constipation
Heah et al. 1997 [17]	Singapore	Non-RCT, single-group (prospective)	16 (16)	0/16 (post treatment); n.a. (later f/u)	Levator ani syndrome	Although BFB had a negligible effect on anorectal physiologic measurements, it was effective in pain relief, with no side effects
Ger et al. 1993 [18]	USA	Non-RCT, 3 arm, non-randomized (prospective)	14 (60)	22/60	Chronic intractable rectal pain ± coccygodynia ± constipation/dyschezia (*n* = 34/60)	BFB vs. EGS vs. epidural steroid caudal block: EGS and BFB had almost the same poor results (>50% were refractory); No significant differences in rates of success or failure after any of the 3 therapeutic options, regardless of whether the option was a primary, secondary, or tertiary choice. Associated historic factors or abnormalities in anorectal physiologic studies did not influence results
Gilliland et al. 1997a [19]	USA	Non-RCT (retrospective)	86 (86)	11/86	Chronic intractable rectal pain ± constipation (*n* = 30/86)	EMG-based BFB can produce alleviation of idiopathic rectal pain. Outcome was significantly improved in patients who completed the treatment schedule compared to those who self-discharged. Outcome was not influenced by patients’ ages, duration of symptoms or prior history of surgery and was not significantly related to the presence of paradoxical puborectalis contraction (EMG or defecography)
Grimaud et al. 1991 [20]	France	Non-RCT, single-group (prospective) + cross-sectional	12 (24) 12 healthy	0/12	Chronic idiopathic anal pain ± constipation (*n* = 9/12)	Chronic idiopathic anal pain is associated with abnormal anorectal manometric profiles (↑ anal canal resting pressure), probably resulting from a dysfunctioning of the striated external anal sphincter. BFB is an effective treatment for chronic idiopathic anal pain. Anorectal pain disappeared after a mean of 8 BFB sessions
**Constipation, dyssynergic defecation**						
Chiarioni et al. 2006 [21]	Italy	RCT (2 arm: intervention vs. different intervention)	54 (109)	14/109 (10/54 in BFB group)	Normal transit constipation due to PFD (Rome II criteria)	5 × 30min BFB sessions are more effective than continuous polyethylene glycol in PF dyssynergia (major improvement in 80%), benefits last at least 2 years. BFB should become the treatment of choice PFD. Predictors of better response to BFB: sensation of incomplete or blocked evacuation, straining with bowel movements; predictors of poorer response: digital facilitation of defecation
Koutsomanis et al. 1994 [22]	Italy	Non-RCT, single-group (prospective)	54 (109)	10/30	Idiopathic constipation	~50% of patients were helped by 2–6 BFB sessions, improvement persisted for ≥ 6–12 months. Both types of PF incoordination (inability to relax on defecation and inability to strain effectively) improved. No clear correlation between change in transit rate and symptomatic outcome
Chiotakakou-Faliakou et al. 1998 [23]	UK	Non-RCT (retrospective)	30 (30)	0/100	Chronic idiopathic slow and normal transit constipation	BFB is an effective long-term treatment for the majority of patients with idiopathic constipation unresponsive to traditional treatment (>50% improved). Patients with slow/normal transit, males/females, with/without paradoxical PF contraction benefited equally. Anorectal testing did not predict outcome
Battaglia et al. 2004 [24]	UK	Non-RCT, single-group (prospective)	100 (100)	n.a.	Chronic constipation (Rome II criteria)	Patients with PFD are likely to have continued benefit from BFB, whereas its effects on slow-transit constipation seems to be maximal in short-term course. Anorectal manometric variables remained unchanged (apart from sensation threshold ↓ in PFD group, maximum rectal tolerable volume ↓ in slow-transit group)
Wang et al. 2003 [25]	Italy	Non-RCT, single-group (prospective)	24 (24)	n.a.	Chronic idiopathic constipation (Rome II criteria)	BFB has a long-term effect with no side effects for most patients (62.5%) with chronic idiopathic constipation unresponsive to traditional treatment. Patients with slow/normal transit, with/without paradoxical PF contraction benefited equally. The psychological status rather than anorectal test could predict outcome. The efficacy of the two modes of BFB was similar
Ba-Bai-Ke-Re et al. 2014 [26]	China	RCT (2 arm: 2 different interventions)	50 (50)	0/44	Chronic obstructive constipation (Rome III criteria)	Manometric BFB-guided PF exercise is superior to oral polyethylene glycol for obstructive defecation for improving overall symptoms, pain at defecation, quality of life
Roy et al. 2000 [27]	China	Non-RCT (retrospective)	44 (88)	n.a.	Chronic idiopathic constipation (no surgery: *n* = 25/78, hysterectomy and no change in bowel function: *n* = 27/78, hysterectomy subjectively led to constipation: *n* = 26/78)	The majority of patients complaining of constipation induced by hysterectomy subjectively respond to behavioral treatment, in a similar proportion to those with idiopathic constipation. Physiological testing did not predict outcome
Chiarioni et al. 2005 [28]	UK	Non-RCT, single group (prospective)	78 (78)	7/52	Chronic idiopathic constipation (PFD: *n* = 34/52, slow transit only: *n* = 12/52, 1–2 criteria for PFD: *n* = 6/52)	BFB is an effective treatment for PFD but not slow-transit constipation. Improvements were maintained at f/u 24 months. BFB eliminated dyssynergia in 91% and enabled 85% to defecate the balloon. Success was predicted by PFD, milder constipation, and less frequent abdominal pain at baseline
Zhu et al. 2011 [29]	Italy	Non-RCT single group, observational	41 (41)	5/41	Functional constipation with PFD (Rome III criteria)	Before treatment, 7 Short Form-36 subscales (except bodily pain) were significantly lower in people with PFD than in healthy individuals. After BFB, all subcategories except general health showed improvement surpassing pretreatment baseline values and equalling those for normal. The total Patient Assessment of Constipation Quality of Life Questionnaire score also dramatically improved as did all subscales
Gilliland et al. 1997b [30]	USA	Non-RCT (retrospective)	194 (194)	16/194	Chronic constipation ± concomitant rectal pain (*n* = 30/194)	Success rate of BFB for constipated patients is less than previously reported (35% complete success, 13% partial success). Success rate ↑ after ≥ 5 sessions and was significantly related to patient’s willingness to complete treatment and number of sessions attended. Neither patient age, sex, abnormalities in manometry nor duration of symptoms significantly affected outcome
Parker et al. 2019 [31]	Canada	Non-RCT (retrospective)	130 (130)	38/168	Chronic constipation + PFD (*n* = 53/130), without PFD (*n* = 3/130), fecal incontinence (*n* = 49/130), constipation + fecal incontinence (*n* = 22/130), rectal pain (*n* = 3/130)	In patients with chronic constipation due to PFD, overall response rate was 69.8%, only 45.3% had symptomatic improvement. These patients are less likely to have symptomatic response than those with fecal incontinence. 3 BFB sessions are insufficient to manage dyssynergic defecation
**Male chronic pelvic pain syndrome, urological chronic pelvic pain syndrome**						
Clemens et al. 2000 [32]	–	Non-RCT, single group	19 (19)	3/19	Nonbacterial male CPPS [33] (NIH type IIIA prostatitis: *n* = 6/19, NIH type IIIB prostatitis: *n* = 13/19)	A formalized program of neuromuscular reeducation of PF muscles with interval bladder training can provide significant, durable improvement in objective measures of pain, urgency, and frequency in patients with CPPS. Detrusor instability, hypersensitivity to filling, or bladder-sphincter pseudodyssynergia on pretreatment urodynamic studies were not predictive of treatment results
Cornel et al. 2005 [34]	USA	Non-RCT, single group; observational	33 (33)	2/33 (15/33 for EMG values)	Male CPPS (NIH type III prostatitis) [33]	BFB physical therapy and PF reeducation lead to a significant symptom improvement and decrease of PF muscle tonus
Yang et al. 2017 [35]	Netherlands	Non-RCT (retrospective)	22 (50)	5/50	Male CPPS (NIH type IIIA, IIIB prostatitis) [33]	Both electromagnetic stimulation and EGS + BFB physical therapy of PF muscle effectively reduce pain, increase quality of life and improve urinary tract symptoms in refractory male CPPS. EGS + BFB had additional benefits on pain and QoL compared to electromagnetic stimulation alone
He et al. 2010 [36]	Taiwan	Non-RCT (retrospective)	21 (21)	n.a.	Nonbacterial male CPPS + dysfunctional voiding	BFB had satisfactory short-term effects on patients with dysfunctional voiding and chronic prostatitis. Urodynamics could be used to help in the diagnosis and select the most appropriate treatment
**Female chronic pelvic pain**						
Schmitt et al. 2017 [37]	USA	Non-RCT, single-group (prospective)	94 (94)	0/94 dropouts, but missing outcome data (*n* : Tables 2, 3 and 4)	Pelvic pain or dyspareunia (*n* = 29/94), defecatory smptoms (*n* = 31/94), urinary symptoms (*n* = 84/94)	An aggressive PF rehabilitation program including BFB with vaginal EGS had a high rate of self-reported subjective success and satisfaction in patients with PF dysfunction
Glazer et al. 1995 [38]	USA	Non-RCT, single-group (prospective)	33 (33)	n.a.	Vulvar vestibulitis syndrome	PF muscle instability is a critical factor in pain associated with vulvar vestibulitis syndrome. A BFB-assisted exercise program that stabilizes PF muscles significantly reduces and, in some cases, eliminates symptoms. The more the PF muscle stabilized, the more pain decreased, the higher the initial pain, the higher the pain reduction. 6‑month f/u indicated maintenance of therapeutic benefits
McKay et al. 2001 [39]	USA	Non-RCT, single-group (prospective)	29 (29)	Monthly f/u, 25/29 (!) after 11 months	Moderate—severe vulvar vestibulitis syndrome	EMG BFB of PF is an effective approach to vulvar vestibulitis. 88.9% reported negligible or mild pain after treatment
Gentilcore-Saulnier et al. 2010 [40]	Canada	Non-RCT, single-group (prospective) + cross-sectional	11 (22)	n.a.	Provoked vestibulodynia ± constipation, dysmenorrhea, urinary frequency	Women with provoked vestibulodynia showed altered PF muscle behavior compared to controls, providing empirical evidence of PF muscle dysfunction, especially at the superficial layer. A physiotherapy rehabilitation program targeting PF muscle dysfunction normalized PF muscle behavior
Bendana et al. 2009 [41]	USA	Non-RCT (retrospective)	52 (52)	0/52 dropouts^a^	Painful PF spasm + urinary urgency/frequency	Combined transvaginal BFB, electrostimulation and behavioral therapy targeting PF relaxation demonstrated statistically significant improvement in urinary symptoms for up to 3 months. Further study for refractory patients is warranted
Philips et al. 1992 [42]	Canada	RCT (randomized yoked design, intervention vs. different vs. no intervention)	10 (30)	n.a.	Urethral syndrome + functional urinary incoordination (pelvic pain: *n* = 11/30, dyspareunia: *n* = 9/30)	Both EMG BFB and progressive muscle relaxation proved effective in improving symptomology and psychological state in patients with functional urinary incoordination
Hart et al. 1981 [43]	USA	Non-RCT, 2 arm: 2 different interventions (prospective)	14 (14)	3/14	Spasmodic dysmenorrhea according to MSQ	BFB and temperature training are effective in reducing many of the painful symptoms of primary dysmenorrhea. There was no significant difference between EMG and temperature training
Bennink et al. 1982 [44]	USA	RCT (3 arm: intervention ± different intervention vs. no intervention)	5 (15)	0/15	Primary dysmenorrhea (spasmodic or congestive)	Subjective reports indicated that the symptoms of dysmenorrhea improved for the BFB group but not for the relaxation or control groups
Vagedes et al. 2019 [45]	Germany	RCT (3 arm: 2 different interventions vs. standard care)	20 (60)	12/60 (6/20 in BFB group)	Primary dysmenorrhea	Preliminary evidence suggests that rhythmical massage might improve pain intensity after 12 weeks compared to usual care. No significant differences were found between heart rate variability-based home-BFB and the control group
Starr et al. 2013 [46]	USA	Non-RCT (retrospective)	778 (778)	97/778	PF dysfunction (urinary: *n* = 694/778, defecatory: *n* = 187/778, pelvic pain: *n* = 368/778)	Comprehensive PF rehabilitation including PF muscle training, BFB, EGS, constipation management, behavioral modification, incontinence devices, and pharmacotherapy is effective in treating women with PF dysfunction
Lúcio et al. 2014 [47]	Brazil	RCT, (3 arm: multimodal + sham vs. local vs. distal electrotherapy)	30 (30)	10/30	Woman with multiple sclerosis + sexual dysfunction	PF muscle training with EMG BFB—alone or combined with intravaginal EGS or transcutaneous tibial nerve stimulation—contributes to the improvement of sexual dysfunction in patients with multiple sclerosis
Aalaie et al. 2020 [48]	Iran	RCT (2 arm: 2 different interventions)	11 (22)	1/22 (in BFB group)	Female sexual pain dysfunction (DSM‑5 criteria [49, 50], FSFI) + stress urinary incontinence	Both BFB and EGS increased the FSFI score. Both interventions decreased pain during vaginal penetration similarly. To improve sexual function, women undergoing BFB seem to benefit more than those receiving EGS
**Chronic pelvic pain in children**						
Hoebeke et al. 2004 [51]	Belgium	Non-RCT, single-group (prospective)	21 (21)	n.a.	PF spasms ± detrusor hyperactivity (*n* = 13/21), ±dysfunctional voiding (*n* = 5/21), ±constipation (*n* = 8/21)	Pelvic floor spasms in children (which can be secondary to detrusor overactivity) respond well to pelvic floor relaxation therapy
Ebiloglu et al. 2016 [52]	Turkey	Non-RCT (retrospective)	136 (136)	n.a.	Overactive bladder syndrome	BFB can be thought of as the first-line treatment option when standard urotherapy fails. Success rate was 53% for urgency, 69% for dysuria. The mean LUTSS significantly improved after BFB. Patients without holding maneuvers, daytime incontinence and enuresis had better recovery compared to the opposites
Ergin et al. 2016 [53]	Turkey	Non-RCT, single-group (prospective) + cross-sectional	52 (100) 48 healthy	13/52	Dysfunctional voiding (*n* = 52/100), +overactive bladder syndrome (*n* = 27/52)	Urinary nerve growth factor (UNGF) Ievels were higher in children with dysfunctional voiding and decreased after BFB. UNGF Ievels could be used for the diagnosis and the assessment of BFB success in these children
Li et al. 2006 [54]	China	Non-RCT, single-group (prospective) + cross-sectional	25 (40) 15 healthy	0/25	Pubertal chronic prostatitis, NIH type II (*n* = 1/25), IIIA (*n* = 3/25), IIIB (*n* = 21/25)	The main type of chronic prostatitis during puberty is IIIB; the dominating symptom is a voiding disorder. The impact on life and psychological effects are substantial. Pubertal boys with chronic prostatitis have PF dysfunction and several abnormal urodynamic values. The effect of BFB in pubertal chronic prostatitis is satisfactory
**Musculoskeletal, low back pain, myofascial pain**						
Kent et al. 2015 [55]	Denmark	RCT (2 arm: intervention + standard care vs. placebo + standard care)	58 (112)	19/112	Subacute—chronic low back pain	Individualized movement retraining using motion-sensor biofeedback resulted in significant and sustained improvements in low back pain. This pilot trial also refined the procedures and sample size requirements for a fully powered RCT

*BFB* biofeedback, *CPPS* chronic pelvic pain syndrome, *CP/CPPS* chronic prostatitis/chronic pelvic pain syndrome, *DSM-5 criteria* diagnostic and statistical manual of mental disorders, *EGS *electrogalvanic stimulation, *EMG* electromyography, *FSFI* female sexual function index [56], *f/u* follow-up, *IG* intervention goup, *LUTSS* lower urinary tract symptom score [57], *MSQ* menstural Symptom questionnaire (MSQ) [58], *NIH* National Institute of Health, *NIH-CPSI* National Institute of Health – chronic prostatitis symptom index score, *PF* pelvic floor, *PFD* pelvic floor dyssynergia, ↓ significant decrease, ↑ significant increase, *RCT* randomized controlled trial, *vs*. versus

^a^missing outcome data: *n* = 31/52 for American Urological Association (AUA) symptom score [59], *n* = 39/52 for visual analog scale (VAS) [60]) symptom severity/effect daily life

**Table 3:** Patient characteristics

Study	Subgroups according to physiological testing (if applicable)	Mean symptom duration ± SD (range)	Sex %		Mean age IG in years ± SD (range)
			Male	Female	
**Anorectal pain syndrome**					
Chiarioni et al. 2010 [16]	n.a.	17.1 ± 4.3 months (“high likely” LAS), 18.6 ± 4.8 (“possible” LAS)	19% (“high likely” LAS), 33% (“possible” LAS)^b^	48%	41.0 ± 10.0 in “high likely” LAS, 41.4 ± 10.3 in “possible” LAS^b^
Heah et al. 1997 [17]	n.a.	32.5 ± 6.7 months	56.3%^b^	43.6%^b^	50.5 (39–66)
Ger et al. 1993 [18]	n.a.	54 (2–228) months	42.9%^b^	57.1%^b^	71 (n.a.)^b^
Gilliland et al. 1997a [19]	High resting pressures (manometry): *n* = 5/14, nonrelaxation or paradoxical PF contraction: *n* = 7/14, abnormal cinedefecography: *n* = 14/14	36 (3-lifelong) months	36.0%^b^	64.0%^b^	68 (12–96)^b^
Grimaud et al. 1991 [20]	n.a.	8 ± 6 (3–72) months	66.7%^b^	33.3%^b^	54 ± 3 (24–66)^b^
**Constipation, dyssynergic defecation**					
Chiarioni et al. 2006 [21]	Slow transit constipation was excluded	>12 months	5.6%^b^	94.4^b^	33.3 ± 1.5^b^
Koutsomanis et al. 1994 [22]	Slow transit: *n* = 2/20, slow transit + pelvic incoordination: *n* = 11/20, pelvic incoordination: *n* = 7/20	n.a.	90.0%^b^	10.0%^b^	34 (18–53)^b^
Chiotakakou-Faliakou et al. 1998 [23]	Slow transit: *n* = 18/100, slow transit + paradoxcial PF contraction: *n* = 29/100, normal transit: *n* = 11/100, normal transit + paradoxical PF contraction: *n* = 15/100	n.a., median age of onset: 21 (0–70)	13.0%^b^	87.0%^b^	40 (10–79)^b^
Battaglia et al. 2004 [24]	PF dyssynergia, *n* = 14/24, slow transit: *n* = 10/24	>12 months	16.7%^b^	83.3%^b^	n.a., (27–54)^b^
Wang et al. 2003 [25]	Slow transit: *n* = 8/50, anorectic outlet obstruction: *n* = 36/50, both: *n* = 6/50	55.2 (30–360) months	28.0%^b^	72.0%^b^	52.6 (16–71)^b^
Ba-Bai-Ke-Re et al. 2014 [26]	n.a.	42 months	n.a.	n.a.	54 (n.a.)
Roy et al. 2000 [27]	Rectal prolapse: *n* = 12/78, rectocele: *n* = 22/78, slow transit: *n* = 53/78, paradoxical PF contraction: *n* = 40/78	Several years	0%^b^	100%^b^	n.a. (24–75)^b^
Chiarioni et al. 2005 [28]	n.a.	168 (24–480) months	05.8%^b^	94.2%^b^	34.9 ± 10.2 (23–63)^b^
Zhu et al. 2011 [29]	n.a.	n.a.	38.9%^b^	61.1%^b^	46.4 (21–65)^b^
Gilliland et al. 1997b [30]	Fixed or dynamic descent: *n* = 100/194, rectocele: *n* = 32/194, intussusception: *n* = 15/194	168 (2–lifelong) months	30.4%^b^	69.6%^b^	71 (11–96)^b^
Parker et al. 2019 [31]	Chronic constipation + dyssynergic defecation *n* = 53/130, without: *n* = 3/130	n.a.	20.8%^b^	79.2%^b^	57.5 ± 16.4^b^
**Male chronic pelvic pain syndrome, urological chronic pelvic pain syndrome**					
Clemens et al. 2000 [32]	n.a.	n.a.	100%^b^	0%^b^	38 (18–67)^b^
Cornel et al. 2005 [34]	Detrusor instability: *n* = 5/19, diminished bladder capacity: *n* = 4/19, dysfunctional voiding of bladder (pseudodyssynergia): *n* = 6/19, cystometric abnormalities + pseudodyssynergia *n* = 3/19	≥3 months	100%^b^	0%^b^	45 (23–70)^b^
Yang et al. 2017 [35]	n.a.	30.4 (6–144) months	100%^b^	0%^b^	43.4 (24–68)^b^
He et al. 2010 [36]	n.a.	>3 months	100%^b^	0%	n.a.
**Female chronic pelvic pain**					
Schmitt et al. 2017 [37]	n.a.	n.a.	0%	100%	52.2 ± 15.4 in *n* = 29 with pelvic pain
Glazer et al. 1995 [38]	n.a.	40.8 (24–72) months	0%	100%	31.5 (21–45)
McKay et al. 2001 [39]	n.a.	44.4 (24–60) months	0%	100%	35 (25–48)
Gentilcore-Saulnier et al. 2010 [40]	n.a.	48 ± 12 months	0%	100%	22 ± 2
Bendana et al. 2009 [41]	n.a.	n.a.	0%	100%	45.0 ± 17, (19–76)
Philips et al. 1992 [42]	n.a.	57 (4–240) months	0%^b^	100%^b^	25.7 ± 4.7^b^
Hart et al. 1981 [43]	n.a.	n.a.	0%	100%	26 ± 6.2
Bennink et al. 1982 [44]	n.a.	n.a.	0%^a^	100%^a^	19.2 (n.a.)^a^
Vagedes et al. 2019 [45]	n.a.	>1 year	0%^a^	100%^a^	29.7 ± 8.0^a^
Starr et al. 2013 [46]	n.a.	n.a.	0%^b^	100%^b^	51 (18–95)^b^
Lúcio et al. 2014 [47]	n.a.	4.1 (0.7–10) months	0%^b^	100%^b^	44.5 (36–51)^b^ in intervention group 1
Aalaie et al. 2020 [48]	n.a.	n.a.	0%	100%	50.7 ± 6.1
**Chronic pelvic pain in children**					
Hoebeke et al. 2004 [51]	n.a.	3 months	9.5%	90.5%	8.3 (n.a.)
Ebiloglu et al. 2016 [52]	Overactive bladder syndrome + dysfunctional voiding: *n* = 107/136, overactive bladder syndrome only: *n* = 29/136	n.a.	29.0%^b^	71.0%^b^	8.11 (5–14)^b^
Ergin et al. 2016 [53]	n.a.	n.a.	23.1%^b^	76.9%^b^	8.84 ± 2.54^b^
Li et al. 2006 [54]	n.a.	>3 months	100%^b^	0%	16.5 ± 1.1 (15–18)^b^
**Musculoskeletal, low back pain, myofascial pain**					
Kent et al. 2015 [55]	n.a.	13 (4.25–13) months	48.0^b^	52.0%^b^	39 ± 12^b^

*LAS* levator ani syndrome, *n.a.* data not available, *PF* pelvic floor, *IG* intervention group, *BFB* biofeedback

^a^all study patients

^b^patients in IG receiving BFB

**Table 4:** Intervention characteristics

Study	Interventions performed in longitudinal study arm	Time points of follow-up	BFB device details	BFB treatment details: number of sessions, duration per session (min/, frequency) (times/week), duration of whole intervention (weeks, if available)	Setting of BFB intervention (clinical/home-based), home exercise (=HE) encouraged (yes/not addressed)
**Anorectal pain syndrome**					
Chiarioni et al.2010 [16]	IG 1: BFB + psychological counselling (5 sessions BFB + 4 sessions psychological counselling) (*n* = 52)	Baseline, 1, 3, 6, 12 months	Anal sEMG probe	5 sessions, 30 min, 1 ×/week	Clinical
	IG 2: EGS (9 sessions, 30–45/min, 3 ×/week) + 4 sessions psychological counselling (*n* = 52)	Baseline, 1, 3, 6, 12 months	–	–	–
	IG 3: massage of levator ani muscle (9 sessions, 30–45 min, 3 ×/week) + 4 sessions psychological counselling (*n* = 53)	Baseline, 1, 3, 6, 12 months	–	–	–
Heah et al. 1997 [17]	BFB	Baseline—2 weeks after treatment, mean f/u 12.8 ± 2.6 months	Anorectal manometry (rectal balloon)	4 sessions, 60 min, 1 ×/week	Clinical (HE: yes)
Ger et al. 1993 [18]	IG 1: EGS (3 sessions, 30–60 min, 7–10 days) (some had failed BFB, epitural caudal block earlier)	Baseline—mean f/u 15 [2–36] months after treatment	–	–	–
	IG 2: BFB (50% had failed EGS earlier)	Same as IG1	Anal sEMG probe	≥6 sessions, 30–60 min, 1 ×/week	Clinical (HE: yes)
	IG 3: epidural steroid caudal block (some had failed other modalities earlier)	Same as IG1	–	–	–
Gilliland et al. 1997a [19]	BFB including education, stress management and cognitive-behavioral psychotherapy techniques	Baseline—after treatment (time-points varied)	Anal sEMG probe	2–18 sessions (until improvement/persistent failure/self-discharge), 60 min; frequency n.a.	Clinical (HE: yes)
Grimaud et al. 1991 [20]	BFB + education	Baseline—after treatment, long term f/u after 16 ± 1 [10–24] months	Anorectal manometry	5–13 sessions, 30 min, 1 ×/week until pain disappearance (8 ± 1 [5–13] weeks), reeducation sessions over 6 months	Clinical (HE: yes)
**Constipation, dyssynergic defecation**					
Chiarioni 2006 [21]	IG1: BFB	Baseline, 6, 12, 24 months after starting treatment	Anal sEMG probe	5 sessions, 30 min, 1 ×/week	Clinical, laxatives at home
	IG2: laxatives (polyethylene glycol 1–2 packets daily + counselling with physician)	Same as IG1	–	–	–
Koutsomanis et al. 1994 [22]	BFB	Baseline, after treatment, 6 weeks after starting treatment, 6–12 months after 6‑week-f/u	sEMG skin electrodes close to anal verge (external anal sphincter), visual + acoustic feedback	Mean 4 [2–6] sessions (until improvement or persistent failure), 30–45 min, 1 ×/week	Clinical (HE: n.a.)
Chiotakakou-Faliakou et al. 1998 [23]	BFB + education, balloon defecation training	Baseline, after treatment, long term f/u (mean 23.4 [12–44]) months after treatment	sEMG skin electrodes close to anal verge (external anal sphincter), visual feedback	Mean 4 [1–10] sessions, duration n.a., 1 ×/1–2 weeks	Clinical (HE: yes)
Battaglia et al. 2004 [24]	BFB + balloon defecation training	Baseline, 3 months, 1 year after treatment	Anal sEMG plug	8 sessions, duration n.a., 2 ×/week, over 4 weeks	Clinical (HE: yes)
Wang et al. 2003 [25]	BFB: EMG vs. manometry based BFB	Baseline, after treatment, long term f/u (mean18 [12–28] months after treatment)	EMG based BFB: surface sEMG electrodes (anal sphincter), auditory + visual feedback; manometry based BFB: visual BFB	5 sessions, 30 min, 1 ×/week	Clinical (HE: yes)
Ba-Bai-Ke-Re et al. 2014 [26]	IG1: BFB	Baseline, 1, 3, 6 months after treatment	Anorectal manometry	4–5 sessions, duration n.a., 1/1–2 week	Clinical (HE: yes)
	IG2: laxatives (polyethylene glycol, 17 g 3 ×/day, 2 weeks)	–	–	–	–
Roy et al. 2000 [27]	BFB	Baseline, after treatment, long-term f/u (mean 28 [12–44] months after treatment)	sEMG skin electrodes close to anal verge (external anal sphincter), visual feedback	4–5 sessions, duration n.a. 1/1–2 week	Clinical (HE: n.a.)
Chiarioni et al. 2005 [28]	BFB + balloon defecation training	Baseline, 1, 6, 12, 24 months after treatment	Anal sEMG plug, visual feedback	5 sessions, 30–45 min, 1 ×/week	Cinical (HE: n.a.)
Zhu et al. 2011 [29]	BFB	Baseline—after treatment (n.a.)	Water-perfused intra-anal instrument, visual + verbal feedback	6–10 sessions, 30–60 min, frequency n.a., over 4–8 weeks	Clinical (HE: yes)
Gilliland et al. 1997b [30]	BFB (+education, stress management, lifestyle modification)	Baseline—after treatment (n.a.)	Anal sEMG probe	2–>30 sessions (until symptom resolution/control over PF muscles in EMG/self-discharge: mean self-discharged: 5, finished: 11), 60 min, further data n.a	Clinical (HE: yes)
Parker et al. 2019 [31]	BFB (+education, exercise instructions, diet)	Baseline—after treatment (n.a.)	Anorectal manometry, visual feedback	Mean 2.9 [2–3] sessions, further data n.a.	Clinical (HE: yes)
**Male chronic pelvic pain syndrome, Urological Chronic Pelvic Pain Syndrome**					
Clemens et al. 2000 [32]	BFB (PF reeducation + bladder training)	Baseline—mean 5.8 [1.6–14.8] months after treatment	sEMG electrodes	≤6 sessions, 60 min, 1 ×/2 week	Clinical (HE: yes)
Cornel et al. 2005 [34]	BFB	Baseline—after treatment (n.a.)	Anal sEMG probe	6–8 sessions, 1 ×/week, later 1 ×/2–4 weeks, duration n.a.	Clinical (HE: n.a.)
Yang et al. 2017 [35]	IG 1: EGS + BFB	Baseline—12 weeks after treatment	Anal sEMG probe	8 sessions, 45 min (15 min BFB, 30 min EGS), 1–2 ×/week, over 6 weeks	Clinical (HE: n.a.)
	IG 2: electomagnetic stimulation (18 sessions, 30 min, 3 ×/week, 6 weeks)	Baseline—12 weeks after treatment	–	–	–
He et al. 2010 [36]	BFB	Baseline—10 weeks after treatment	Anal sEMG probe	No. sessions n.a., 30 min, 2–3 ×/week, over several weeks	Clinical (HE: n.a.)
**Female chronic pelvic pain**					
Schmitt et al. 2017 [37]	BFB + vaginal EGS + behavioral modification + pharmacologic therapies for urinary and defecatory management	Baseline, after 1st, 3rd, final treatment session	sEMG skin electrodes (abdominals), vaginal/rectal sEMG probe	4–7 sessions (until ≥ 80% improvement), BFB + 30 min vaginal EGS, 1 ×/2 week	Clinical (HE: yes)
Glazer et al. 1995 [38]	BFB	Baseline, f/u at 6 clinical evaluation appointments + 6 months after 6th reevaluation	sEMG portable vaginal probe, visual feedback	20 min, 2 ×/day, 7 ×/week, after 6 evaluations: exercises continued without BFB ≥ 3 months	Home-based, 6 × clinical f/u
McKay et al. 2001 [39]	BFB	Baseline, f/u every 4 weeks	sEMG portable vaginal probe, visual feedback	No. sessions n.a., duration n.a., 60 repetitions, 2 ×/day, 7 ×/week, up to 11 months	Home-based, 1 ×/4 weeks clinical f/u
Gentilcore-Saulnier et al. 2010 [40]	BFB + education, manual therapy, EGS, dilator insertion	Baseline—after treatment (n.a.)	sEMG vaginal probe (deep PF), sEMG electrodes (superficial PF)	8 sessions, 60–75 min overall (10–15 min BFB), frequency n.a, over 12 ± 3 weeks	Clinical (HE: yes)
Bendana et al. 2009 [41]	BFB + education, vaginal EGS	Baseline, after treatment, 3 months after treatment	sEMG vaginal probe	6 sessions, 60 min (10 min BFB, 20 min EGS), 1 ×/week	Clinical (HE: n.a.)
Philips et al. 1992 [42]	IG1: BFB (±retention control/pain management techniques)	Baseline, after treatment, 2 months after treatment	Perivaginal sEMG electrodes, visual feedback	Mean 8 [5–12] sessions (until aim reached), further data n.a.	Clinical (HE: yes)
	IG2: progressive muscle relaxation (±retention control/pain management), session number same as yoked partner in BFB group	Same as IG1	–	–	–
	Comparison group: no intervention (cross-over after 2 months)	Same as IG1	–	–	–
Hart et al. 1981 [43]	IG 1: EMG general relaxation BFB	Baseline, after treatment (8 weeks), 8 weeks after treatment	sEMG electrodes frontalis muscle, aural feedback	Mean: 12.9 [9–15] sessions, 30 min, 2 ×/week, over 2 menstrual cycles	Clinical (HE: yes)
	IG2: temperature general relaxation BFB	Same as IG1	Skin temperature, visual + aural feedback	Same as IG1	Same as IG1
Bennink et al. 1982 [44]	IG1: BFB + general relaxation	Baseline (interview), after first menstrual cycle (before treatment) and ~1 week after 3rd or 4th cycle (post treatment)	sEMG electrodes (lower abdomen), aural feedback	5 sessions, 30 min, 3 sessions before, 2 sessions on first 2 days of period	Clinical (HE: yes)
	IG2: same general and PF relaxation training without BFB	Same as IG1	–	–	–
	CG: no intervention	Same as IG1	–	–	–
Vagedes et al. 2019 [45]	IG1: BFB—slow breathing technique (general relaxation)	Baseline—after treatment (n.a.)	Heart rate variability Qiu (Biosign) device, visual feedback	15 min/day, 7 ×/week, over 12 weeks	Home-based, clinical f/u after 1, 3, then every 4 weeks
	IG2: rhythmical massage (anthroposophic medicine) 30–45 min, 1 ×/week, 3 months	Same as IG1	–	–	–
	CG: standard care (analgesics, physical exercise, warmth)	Same as IG1	–	–	–
Starr et al. 2013 [46]	Complex PF rehabilitation: instruction, behavioral management, EGS, BFB	2^nd^ BFB treatment—after treatment	sEMG electrodes (abdominals), vaginal sEMG probe, anorectal manometry	5–8 sessions (8 if improvement < 80% after 5 sessions), 1 ×/2 weeks	Clinical (HE: yes)
Lúcio et al. 2014 [47]	IG1: BFB + PF muscle training + placebo EGS	Baseline—after treatment (12 weeks)	sEMG vaginal probe	24 sessions, 30 min, 2 ×/week, 12 weeks	Clinical (HE: yes)
	IG2: BFB + PF muscle training + vaginal EGS (30 min, 2 ×/week, 12 weeks)	Same as IG1	Same as IG1	Same as IG1	Same as IG1
	IG3: BFB + PF muscle training + transcutaneous tibial nerve stimulation (30 min, 2 ×/week, 12 weeks)	Same as IG1	Same as IG1	Same as IG1	Same as IG1
Aalaie et al. 2020 [48]	IG1: BFB, 100 min, 2 ×/week, 6 weeks + Kegel exercises at home	Baseline—2, 3 months after treatment	sEMG vaginal probe	12 sessions, 100 min, 2 ×/week, over 6 weeks	Clinical (HE: yes)
	IG2: vaginal EGS (50 min of stimulation, 2 ×/week, 6 weeks) + Kegel exercises at home	Same as IG1	–	–	–
**Chronic pelvic pain in children**					
Hoebeke et al. 2004 [51]	BFB ± anticholinergics (*n* = 13/21 with detrusor hyperactivity)	Baseline, after treatment (12 weeks)	Anal plug sEMG, visual BFB	12 sessions, duration n.a., 1 ×/week	Clinical (HE: n.a.)
Ebiloglu et al. 2016 [52]	BFB	Baseline, f/u at 3rd and 6th month (total treatment time: 6 months)	Uroflowmeter + sEMG perineal electrodes (external sphincter), visual feedback	4 sessions, 10 min, 1 ×/week (1^st^ month), then continued without BFB, f/u BFB at 3^rd^, 6^th^ month	Clinical (HE: yes)
Ergin et al. 2016 [53]	BFB	Baseline, after treatment (6 months)	Uroflowmetry including sEMG	≥6 sessions, over 6 months, further data n.a.	Clinical (HE: n.a.)
Li et al. 2006 [54]	BFB	Baseline, f/u after ~12 weeks	Urodynamic system: anal sEMG probe, abdominal pressure (intra-anal balloon catheter)	No. sessions n.a., 20–30 min, 2–3 ×/week, over several weeks	Clinical (HE: n.a.)
	CG: healthy controls, no intervention	–	–	–	–
**Musculoskeletal, low back pain, myofascial pain**					
Kent et al. 2015 [55]	IG-BFB: BFB based movement modification + education, guidelines-based medical or physiotherapy care	6 × during 10-weeks of treatment (baseline, week 1, 3, 6, 8, 10), f/u at week 12, 26, 52	Motion-sensor movement biofeedback (ViMove device), sEMG sensors, aural + vibrational feedback	6 (subacute pain)–8 (chronic pain) sessions, over 10 weeks, frequency n.a.	Clinical + home-based
	IG-placebo: placebo + education, guidelines-based medical or physiotherapy care	Same as IG-BFB	–	–	–

*BFB* biofeedback, *CG* control group, *EGS* electrogalvanic stimulation, *EMG* electromyography, *sEMG* surface electromyography, *HE* home exercise, *f/u* follow-up, *IG* intervention group, *min* minute(s), *PF* pelvic floor

Outcome data were presented by means of the mean difference within a study group or between groups and their statistical significance (Tables 5, 6 and 7). Few studies provided effect sizes or the corresponding interval estimates (e.g. the confidence intervals) for the mean differences. These values were calculated by the authors if studies provided the relevant data to do so. The criteria for determining effect sizes according to Cohen [61] are listed in the legend of Tables 5 and 7.

**Table 5:** Primary outcome: effect on pain and overall symptoms

Study	*n* (total) (group 1, 2)	Group 1 (n1)	Group 2 (n2)	Outcome measure	Mean difference: Group 2 minus Group 1 [CI]	Effect size [strength]	*P*-value (for difference in means)
**Anorectal pain syndrome**							
Chiarioni et al. 2010 [16]	104	IG1 (BFB) in pat. w LAS (52)	IG2 (EGS) in pat. w LAS (52)	SR: % pat. w adequate pain relief after 1 month	−26.9	n.a.	***p*** **<** **0.01**^**f**^
	104	IG1 (BFB) in pat. w LAS (52)	IG2 (EGS) in pat. w LAS (52)	SR: % pat. w adequate pain relief after 3 months	−18.9	n.a.	***p*** **<** **0.01**^**f**^
	104	IG1 (BFB) in pat. w LAS (52)	IG2 (EGS) in pat. w LAS (52)	SR: % pat. w adequate pain relief after 6 months	−31.2	n.a.	***p*** **<** **0.01**^**f**^
	104	IG1 (BFB) in pat. w LAS (52)	IG2 (EGS) in pat. w LAS (52)	SR: % pat. w adequate pain relief after 12 months	−31.2	n.a.	***p*** **<** **0.01**^**f**^
	105	IG1 (BFB) in pat. w LAS (52)	IG3 (massage) in patients with LAS (53)	SR: % pat. w adequate pain relief after 1 month	−31.3	n.a.	***p*** **<** **0.01**^**f**^
	105	IG1 (BFB) in pat. w LAS (52)	IG3 (massage) in patients with LAS (53)	SR: % pat. w adequate pain ↓ after 3, 6, 12 months	−36.9	n.a.	***p*** **<** **0.01**^**f**^
	104	BFB in pat. w “high likely” LAS (n.a.)	EGS in pat. w “high likely” LAS (n.a.)	SR: % pat. w adequate pain relief after 1, 3, 6, 12 months	In favour of BFB group	n.a.	***p*** **<** **0.025**^**f**^
	105	BFB in pat. w “high likely” LAS (n.a.)	Massage in pat. w “high likely” LAS (n.a.)			n.a.	***p*** **<** **0.025**^**f**^
	104	BFB in pat. w “possible” LAS (n.a.)	EGS in pat. w “possible” LAS (n.a.)	SR: % pat. w adequate pain relief after 1, 3, 6, 12 months	–	n.a.	*p* > 0.025^f^
	105	BFB in pat. w “possible” LAS (n.a.)	Massage in pat. w “possible” LAS (n.a.)		–	n.a.	*p* > 0.025^f^
	104	BFB in pat. w “high likely” LAS (n.a.)	EGS in pat. w “high likely” LAS (n.a.)	Subjective change in pain to baseline, ordinal scale [−2 to +3: −2 “a lot worse” to +3 “a lot better/cured”] after 1, 3, 6 months	In favour of BFB group	n.a.	***p*** **<** **0.025**^**d**^
	105	BFB in pat. w “high likely” LAS (n.a.)	Massage in pat. w “high likely” LAS (n.a.)			n.a.	***p*** **<** **0.025**^**d**^
	104	BFB in pat. w “possible” LAS (n.a.)	EGS in pat. w “possible” LAS (n.a.)		–	n.a.	*p* > 0.025^d^
	105	BFB in pat. w “possible” LAS (n.a.)	Massage in pat. w “possible” LAS (n.a.)		–	n.a.	*p* > 0.025^d^
	104	BFB in pat. w “high likely” LAS (n.a.)	EGS in pat. w “high likely” LAS (n.a.)	Number of days/months with rectal pain as stated in symptom log (0–30 days) after 1, 3, 6 months	In favour of BFB group	n.a.	***p*** **<** **0.025**^**d**^
	105	BFB in pat. w “high likely” LAS (n.a.)	Massage in pat. w “high likely” LAS (n.a.)			n.a.	***p*** **<** **0.025**^**d**^
	104	BFB in pat. w “possible” LAS (n.a.)	EGS in pat. w “possible” LAS (n.a.)		–	n.a.	*p* > 0.025^d^
	105	BFB in pat. w “possible” LAS (n.a.)	Massage in pat. w “possible” LAS (n.a.)		–	n.a.	*p* > 0.025^d^
	104	BFB in pat. w “high likely” LAS (n.a.)	EGS in pat. w “high likely” LAS (n.a.)	Pain: VAS (0–10 cm), average value of worst pain/wk, after 1, 3, 6 months	In favour of BFB group	n.a.	***p*** **<** **0.025**^**d**^
	105	BFB in pat. w “high likely” LAS (n.a.)	Massage in pat. w “high likely” LAS (n.a.)			n.a.	***p*** **<** **0.025**^**d**^
	104	BFB in pat. w “possible” LAS (n.a.)	EGS in pat. w “possible” LAS (n.a.)		–	n.a.	*p* > 0.025^d^
	105	BFB in pat. w “possible” LAS (n.a.)	Massage in pat. w “possible” LAS (n.a.)		–	n.a.	*p* > 0.025^d^
Heah et al. 1997 [17]	16	Study group pre-BFB (16)	Study group post-BFB (16)	Pain VAS (0–10)	−6	n.a.	***p*** **<** **0.02**
	16	Study group pre-BFB (16)	Study group post-BFB (16)	SR: % pat. needing analgesics	−87.5	n.a.	***p*** **<** **0.03**
Ger et al. 1993 [18]	14	*n*/a	IG2 (BFB group) post-BFB (14)	SR: % pat. w complete pain relief	14.3	n.a.	n.a.
	14	*n*/a	IG2 (BFB group) post-BFB (14)	SR: % pat. w improved pain frequency/intensity	28.6	n.a.	n.a.
	14	*n*/a	IG2 (BFB group) post-BFB (14)	SR: % pat. w no improvement	57.1	n.a.	n.a.
Gilliland et al. 1997a [19]	75	BFB in pat. w rectal pain only (47)	BFB in pat. with rectal pain and constipation (28)	SR: % patients reporting symptom improvement	−4.02	n.a.	*p* = 0.81
	46	BFB subgroup of Group 1: pat. who finished trial (7)	BFB subgroup of Group 1: self-discharged early (39)		−57.5	n.a.	***p*** **<** **0.01**
	28	BFB subgroup of Group 2: pat. who finished trial (n.a.)	BFB subgroup of Group 2: self-discharged early (n.a.)		−46.7	n.a.	***p*** **<** **0.05**
**Constipation, dyssynergic defecation**							
Chiarioni et al. 2006 [21]	54	IG1 (BFB) pre-treatment (54)	IG1 (BFB) 6 months after starting treatment (54)	Frequency of abdominal pain/wk (symptom diary)	−0.69 [−0.74;−0.64]	−5.86 [H]	***p*** **<** **0.01**^**c**^
	54	IG1 (BFB) pre-treatment (54)	IG1 (BFB) 12 months after starting treatment (54)		−0.68 [−0.73; −0.63]	−5.78 [H]	***p*** **<** **0.01**^**c**^
	109	IG1 (BFB) 6 months after starting treatment (54)	IG2 (laxatives) 6 months after starting treatment (55)		0.63 [0.57;0.69]	4.85 [H]	***p*** **<** **0.01**^**c**^
	109	IG1 (BFB) 6 months after starting treatment (54)	IG2 (laxatives) 12 months after starting treatment (55)		0.58 [0.52;0.64]	4.26 [H]	***p*** **<** **0.01**^**c**^
	109	IG1 (BFB) 6 + 12 months after starting treatment (54)	IG2 (laxatives) 6 + 12 months after starting treatment (55)	SR: % patients reporting symptom improvement (4 out of a scale 0–4)	−57.8	n.a.	n.a.
	54	*n*/a	IG1 (BFB) 6 + 12 months after starting treatment (54)		79.6	n.a.	n.a.
	54	*n*/a	IG1 (BFB) 24 months after starting treatment (54)		81.5	n.a.	n.a.
Koutsomanis et al. 1994 [22]	20	Study group pre-BFB (20)	Study group immediately post-BFB (20)	SR: % patients reporting abdominal pain ≥ 1/week	−20	n.a.	≥0.05^b^
	18	Study group pre-BFB (20)	Study group 6 weeks after starting BFB (18)		−13.3	n.a.	≥0.05^b^
	20	Study group pre-BFB (20)	Study group 6–12 months after 6‑wk-f/u (20)		−10	n.a.	≥0.05^b^
	20	Study group pre-BFB (20)	Study group immediately post-BFB (20)	Weekly total pain score (daily pain score: 0 = none, 3 = severe)	−5.5	n.a.	≥0.05^b^
	18	Study group pre-BFB (20)	Study group 6 weeks after starting BFB (18)		−8	n.a.	***p*** **<** **0.01**^**b**^
	20	Study group pre-BFB (20)	Study group 6–12 months after 6‑wk-f/u (20)		−9	n.a.	***p*** **<** **0.01**^**b**^
	20	Study group pre-BFB (20)	Study group immediately post-BFB (20)	Weekly overall symptom score (daily score: 0 = better, 1 = same, 2 = worse)	−4	n.a.	***p*** **<** **0.01**^**b**^
	18	Study group pre-BFB (20)	Study group 6 weeks after starting BFB (18)		−4	n.a.	***p*** **<** **0.01**^**b**^
	20	Study group pre-BFB (20)	Study group 6–12 months after 6‑wk-f/u (20)		−6	n.a.	***p*** **<** **0.01**^**b**^
Chiotakakou-Faliakou et al. 1998 [23]	100	Study group pre-BFB (100)	Study group post-BFB (100)	SR: % patients with abdominal pain	−16	n.a.	***p*** **=** **0.003**^**f**^
	100	Study group pre-BFB (100)	Study group long-term (mean 23.4 months) post-BFB (100)		−20	n.a.	***p*** **=** **0.0004**^**f**^
	100	*n*/a	Study group post-BFB (100)	SR: % patients stating BFB improved bowel symptoms (a little‑a lot)	66	n.a.	n.a.
	100	*n*/a	Study group long-term (mean 23.4 months) post-BFB (100)		55	n.a.	n.a.
	100	*n*/a	Study group post-BFB (100) in pat. w constipation	SR: % patients reporting sonstipation symptom improvement (a little‑a lot)	50	n.a.	n.a.
	100	*n*/a	Study group long-term (23.4 months) post-BFB (100) in pat. w constipation		57	n.a.	n.a.
Battaglia et al. 2004 [24]	14	Subgroup with PF dyssynergia pre-BFB (14)	This subgroup 3 + 12 months after BFB (14)	SR: % patients with abdominal pain	−21.4	n.a.	n.a.
	10	Subgroup with slow transit constipation pre-BFB (10)	This subgroup 3 months post-BFB (10)		−80	n.a.	n.a.
	10	Subgroup with slow transit constipation pre-BFB (10)	This subgroup 12 months post-BFB (10)		−20	n.a.	n.a.
Wang et al. 2003 [25]	50	Study group pre-BFB (50)	Study group post-BFB (50)	SR: % patients with perianal pain at defacation	−28	n.a.	≥0.05^a^
	50	Study group pre-BFB (50)	Study group 1‑year post-BFB (50)		−38	n.a.	≥0.05^a^
	50	*n*/a	Study group post-BFB (50)	SR: % patients reporting overall symptom improvement	62	n.a.	n.a.
	8	*n*/a	Pat. w slow transit constipation post-BFB (8)		37.5	n.a.	n.a.
	36	*n*/a	Pat. w PF dysfunction post-BFB (36)		72.2	n.a.	n.a.
	6	*n*/a	Pat. w combined PF dysf. +slow transit post-BFB (6)		33.3	n.a.	n.a.
Ba-Bai-Ke-Re et al. 2014 [26]	88	IG1 (BFB) 1 month post-treatment (44)	IG2 (laxatives) 1 month post-treatment (44)	SR; % of patients with peri-anal pain at defecation	36.4	n.a.	**0.0006**^**a**^
	88	IG1 (BFB) 3 months post-treatment (44)	IG2 (laxatives) 3 months post-treatment (44)		20.5	n.a.	0.0534^a^
	88	IG1 (BFB) 6 months post-treatment (44)	IG2 (laxatives) 6 months post-treatment (44)		20.5	n.a.	**0.0375**^**a**^
	88	IG1 (BFB) 1 month post-treatment (44)	IG2 (laxatives) 1 month post-treatment (44)	Symptom score: Wexner constipation summary score (0–30 = worst) [62]	−6.00 [−7.41; −4.59]	−1.45 [VL]	***p*** **<** **0.001**^**a**^
	88	IG1 (BFB) 3 months post-treatment (44)	IG2 (laxatives) 3 months post-treatment (44)		−5.00 [−6.21;−3.78]	−1.40 [VL]	***p*** **<** **0.001**^**a**^
	88	IG1 (BFB) 6 months post-treatment (44)	IG2 (laxatives) 6 months post-treatment (44)		−6.00 [−7.11; −4.89]	−1.84 [VL]	***p*** **<** **0.001**^**a**^
Roy et al. 2000 [27]	26	Pre-BFB in pat. w constipation, attributed to hysterectomy by patient (26)	Post-BFB in patients with constipation, attributed to hysterectomy by patient (26)	SR: % of patients with abdominal pain	−23.1	n.a.	n.a.
	26	Pre-BFB in pat. w constipation, attributed to hysterectomy (26)	Long-term (28 months) post-BFB in patients with constipation, attributed to hysterectomy (26)		−11.5	n.a.	n.a.
	27	Pre-BFB in pat. w constipation, not attributed to hysterectomy (27)	Post-BFB in pat. w constipation, not attributed to hysterectomy (27)		−29.6	n.a.	n.a.
	27	Pre-BFB in pat. w constipation, not attributed to hysterectomy (27)	Long-term (28 months) post-BFB in pat. w constipation, not attributed to hysterectomy (27)		−29.6	n.a.	n.a.
	25	Pre-BFB in pat. w constipation, no history of hysterectomy (25)	Post-BFB in pat. w constipation, no history of hysterectomy (25)		−28.0	n.a.	n.a.
	25	Pre-BFB in pat. w constipation, no history of hysterectomy (25)	Long-term (28 months) post-BFB in pat. w constipation, no history of hysterectomy (25)		−36.0	n.a.	n.a.
	78	Pre-BFB in all pat. w constipation (78)	Post-BFB in all pat. w constipation (78)		−27.0	n.a.	n.a.
	78	Pre-BFB in all pat. w constipation (78)	Long-term (28 months) post-BFB in all pat. w constipation (78)		−25.6	n.a.	n.a.
	78	*n*/a	28 months post-BFB in all pat. w constipation (78)	SR: % patients reporting constipation symptom improvement	61.5	n.a.	n.a.
Chiarioni et al. 2005 [28]	41	Subgroup with PF dysfunction after 1, 6, 12, 24 months after BFB (52, 50, 49, 45)	Subgroup with slow transit only, after 1, 6, 12, 24 months after BFB (52, 50, 49, 45)	Pain frequency (in favour of subgroup PF dyssynergia)	n.a.	n.a.	***p*** **<** **0.05**^**a**^
Zhu et al. 2011 [29]	36	Study group pre-BFB (36)	Study group post-BFB (36)	SF-36 subscale pain (0–100: best)	10.3 [−1.31;21.91]	0.48 [S]	***p*** **=** **0.001**^**a**^
	36	Study group pre-BFB (36)	Study group post-BFB (36)	Symptom score (0–15 : 0 = none, 3 = severe for 5 symptoms)	−5.77 [−7.29;−4.25]	−2.04 [H]	***p*** **<** **0.001**^**a**^
Gilliland et al. 1997b [30]	178	*n*/a	Study group post-BFB (178)	SR: % patients with ≥ 3 bowel movements/wk without aid (“complete success”)	35.0	n.a.	n.a.
	178	*n*/a	Study group post-BFB (178)	SR: % patients with < 3 bowel movements/wk with reduced aid (“partial success”)	13.5	n.a.	n.a.
	178	*n*/a	Study group post-BFB (178)	SR: % patients with no improvement (“failed”)	51.1	n.a.	n.a.
	60	*n*/a	Study group post-BFB, pat. attended 2–4 sessions (60)	SR: % patients with ≥ 3 bowel movements/wk without aid (“complete success”)	18.0	n.a.	n.a.
	118	*n*/a	Study group post-BFB, pat. attended ≥ 5 sessions (118)		44.0	n.a.	n.a.
	178	*n*/a	Study group post-BFB, pat. attended 2–4 sessions (60)		−26.0	n.a.	***p*** **<** **0.001**
	52	*n*/a	Study group post-BFB, pat. completed BFB (52)		63.0	n.a.	n.a.
	126	–	Study group post-BFB, pat. not completed BFB (126)		(25.0)	n.a.	n.a.
	178	Study group post-BFB, pat. completed BFB (52)	Study group post-BFB, pat. not completed BFB (126)		−38.0	n.a.	n.a.
Parker et al. 2019 [31]	130	*n*/a	Whole study group post-BFB (130)	SR: % patients reporting symptom improvement (±improvement in anorectal manometry profile)	(55.4)	n.a.	n.a.
	53	*n*/a	Subgroup with constipation + dys. defecation post-BFB (53)		(45.3)	n.a.	n.a.
	3	*n*/a	Subgroup with rectal pain post-BFB (3)		(0.0)	n.a.	n.a.
**Male chronic pelvic pain syndrome, Urological Chronic Pelvic Pain Syndrome**							
Clemens et al. 2000 [32]	16	Study group pre-BFB (19)	Study group 6 months post-BFB (16)	Pain VAS (0–9)	−4	n.a.	***p*** **=** **0.001**^**b**^
	16	Study group pre-BFB (19)	Study group 6 months post-BFB (16)	Symptom score: AUA [59]	−7.5	n.a.	***p*** **=** **0.001**^**b**^
Cornel et al. 2005 [34]	31	Pstudy group re-BFB (33)	Study group post-BFB (31)	Symptom score: NIH-CPSI subdomain pain (0–21)	−5.3	n.a.	***p*** **=** **0.001**^**b**^
	31	Study group pre-BFB (33)	Study group post-BFB (31)	Symptom score: NIH-CPSI	−12.2	n.a.	***p*** **=** **0.001**^**b**^
Yang et al. 2017 [35]	22	IG1 (BFB + EGS) pretreatment (24)	IG1 (BFB + EGS) 12 weeks post-treatment (22)	Pain VAS (0–10)	−3.5 [−4.91;−2.09]	−1.74 [VL]	***p*** **=** **0.001**^**b**^
	45	IG1 (BFB + EGS) 12 weeks post-treatment (22)	IG2 (PEMF) 12 weeks post-treatment (23)		0.6 [−1.44;0.24]	−0.34 [S]	*p* = 0.084^a^
	22	IG1 (BFB + EGS) pretreatment (24)	IG1 (BFB + EGS) 12 weeks post-treatment (22)	NIH-CPSI subdomain pain (0–21)	−8.3 [−10.91;−5.70]	−2.23 [H]	***p*** **<** **0.001**^**a**^
	45	IG1 (BFB + EGS) 12 weeks post-treatment (22)	IG2 (PEMF) 12 weeks post-treatment (23)		0.1 [−1.98;1.78]	−0.03 [VS]	*p* = 0.035^a^
	22	IG1 (BFB + EGS) pretreatment (24)	IG1 (BFB + EGS) 12 weeks post-treatment (22)	Symptom score: NIH-CPSI total score (0–43)	−14.3 [−19.82;−8.78]	−1.81 [VL]	***p*** **<** **0.001**^**a**^
	45	IG1 (BFB + EGS) 12 weeks post-treatment (22)	IG2 (PEMF) 12 weeks post-treatment (23)		0.5 [−3.98;2.98]	−0.07 [VS]	***p*** **=** **0.009**^**a**^
	22	IG1 (BFB + EGS) pretreatment (24)	IG1 (BFB + EGS) 12 weeks post-treatment (22)	Symptom score: IPPS	−4.6 [−8.64;−0.56]	−0.80 [M]	***p*** **=** **0.004**^**a**^
	45	IG1 (BFB + EGS) 12 weeks post-treatment (22)	IG2 (PEMF) 12 weeks post-treatment (23)		1.80 [−3.57;−0.03]	−0.49 [S]	*p* = 0.663^a^
He et al. 2010 [36]	21	Study group pre-BFB (21)	Study group 10 weeks post-BFB (21)	NIH-CPSI subdomain pain (0–21)	−1.80 [−3.13;−0.47]	−0.97 [L]	***p*** **<** **0.05**^**b**^
	21	Study group pre-BFB (21)	Study group 10 weeks post-BFB (21)	Symptom score: NIH-CPSI total score (0–43)	−13.3 [−16.7;−9.92]	−2.83 [H]	***p*** **<** **0.05**^**b**^
**Female chronic pelvic pain**							
Schmitt 2017 et al. [37]	26	Subgroup w pelvic pain/dyspareunia pre-treatment (29)	Subgroup w pelvic pain/dyspareunia after 3rd treatment (26)	Pelvic pain VAS (0–10)	−1	n.a.	*p* = 0.99^b^
	27	Subgroup w pelvic pain/dyspareunia pre-treatment (29)	Subgroup w pelvic pain/dyspareunia after final treatment (27)		−3	n.a.	*p* = 0.27^b^
	26	Subgroup w pelvic pain/dyspareunia after 3rd treatment (26)	Subgroup w pelvic pain/dyspareunia after final treatment (27)		−2	n.a.	***p*** **=** **0.02**^**b**^
	27	Subgroup w pelvic pain/dyspareunia after 3rd treatment (28)	Subgroup w pelvic pain/dyspareunia after final treatment (27)	Rating treatment success pelvic pain (0: none–10: very successful)	2	n.a.	*p* = 0.51^b^
	14	Subgroup w pelvic pain/dyspareunia after 3rd treatment (14)	Subgroup w pelvic pain/dyspareunia after final treatment (16)	Rating treatment success dyspareunia (0: none–10: very successful)	3	n.a.	*p* = 0.20^b^
	79	Subgroup w urinary symptoms after 3rd treatment (80)	Subgroup w urinary symptoms after final treatment (79)	Rating treatment success urinary symptoms (0: none–10: very successful)	2	n.a.	***p*** **<** **0.001**^**b**^
	15	Subgroup w defecatory symptoms after 3rd treatment (16)	Subgroup w defecatory symptoms after final treatment (15)	Rating treatment success defecatory symptoms (0: none–10: very successful)	1	n.a.	*p* = 0.003^b^
Glazer et al. 1995 [38]	33	Study group pre-BFB (33)	Study group after 6th clinical reevaluation with BFB (33)	Pelvic pain VAS (0–10)	−5.7	n.a.	***p*** **<** **0.001**^**b**^
	33	Study group pre-BFB (33)	Study group 6 months after 6th clinical reevaluation (33)		−6	n.a.	***p*** **<** **0.001**^**b**^
	33	Study group pre-BFB (33)	Study group after 6th clinical reevaluation + 6 months later (33)	SR: % of patients reporting intercrouse ≥ 1/month	66.67	n.a.	***p*** **<** **0.001**^**b**^
McKay et al. 2001 [39]	11	Study group 1 month post-BFB (19)	Study group 6 months post BFB (11)	Pelvic pain VAS (0–10)	−6.8	n.a.	n.a.
	11	Study group 1 month post-BFB (19)	Study group 6 months post BFB (11)	SR: % patients reporting intercourse	88.9	n.a.	n.a.
Gentilcore-Saulnier et al. 2010 [40]	11	IG pre-BFB in pat. with provoked vestibulodynia (11)	IG post-BFB in pat. w provoked vestibulodynia (11)	Pain NRS (0–10) during digital intravaginal assessment of superficial + deep PF	−2.00 [−3.33;−0.67]	−1.56 [VL]	***p*** **=** **0.007**^**a**^
	22	IG pre-BFB in pat. w provoked vestibulodynia (11)	Healthy CG without intervention (11)		−2.13 [−1.27;−2.99]	−1.73 [VL]	***p*** **=** **0.002**^**a**^
	22	IG post-BFB in pat. w provoked vestibulodynia (11)	Healthy CG without intervention (11)		−0.13 [−0.26;0.52]	0.23 [S]	*p* = 0.58^a^
	11	IG pre-BFB in pat. w provoked vestibulodynia (11)	IG post-BFB in pat. w provoked vestibulodynia (11)	“unpleasantness” NRS (0–10) during manual assessment	−1.63 [−3.03;−023]	−1.21 [VL]	***p*** **=** **0.0009**^**a**^
	22	IG pre-BFB in pat. w provoked vestibulodynia (11)	Healthy CG without intervention (11)		−0.54 [−0.50;1.58]	0.36 [S]	*p* = 0.40^a^
	22	IG post-BFB in pat. w provoked vestibulodynia (11)	Healthy CG without intervention (11)		−1.09 [−2.01;−0.17]	−0.83 [L]	*p* = 0.07^a^
	11	IG pre-BFB in pat. w provoked vestibulodynia (11)	IG post-BFB in pat. w provoked vestibulodynia (11)	Painful pressure stimulus intensity levels (pressure to induce pain NRS 6/10, g/cm^2^)	92.00 [−162.25;346.25]	0.37 [S]	*p* = 0.07^a^
	22	IG pre-BFB in pat. w provoked vestibulodynia (11)	Healthy CG without intervention (11)		201.00 [−366.64;−35.36]	−0.85 [L]	***p*** **=** **0.001**^**a**^
	22	IG post-BFB in pat. w provoked vestibulodynia (11)	Healthy CG without intervention (11)		109.00 [−246.63;28.63]	−0.56 [M]	***p*** **=** **0.03**^**a**^
Bendana et al. 2009 [41]	21	Study group pre-BFB (52)	Study group 3 months post-BFB (21)	AUA symptom score—total score (0–35)	−7.97 [−12.25;−4.62]	−1.10 [L]	***p*** **<** **0.001**^**a**^
	21	Study group pre-BFB (52)	Study group 3 months post-BFB (21)	American Urological Association bother score (0–6)	−1.53 [−2.33; −0.87]	−1.13 [L]	***p*** **<** **0.001**^**a**^
	13	Study group pre-BFB (52)	Study group 3 months post-BFB (13)	VAS for symptom severity (1 = lowest 10 = most severe)	−2.44 [n.a.]	n.a.	***p*** **<** **0.001**^**a**^
Philips et al. 1992 [42]	10	IG1 (BFB group) pre-BFB (10)	IG1 (BFB group) post-BFB (10)	Pain score: self-monitored	−1.30 [−29.40;26.80]	−0.05 [VS]	n.a.
	10	IG1 (BFB group) pre-BFB (10)	IG1 (BFB group) 2 months post-BFB (10)		−11.40 [−26.55;3.75]	−0.82 [L]	n.a.
Hart et al. 1981 [43]	5	IG1: EMG BFB (5) baseline	IG1: EMG BFB (5) posttreatment	Symptom score: SSS total score: total of 15 symptoms (1 best −5 worst)	−7.00 [−11.85; −2.15]	−2.51 [H]	n.a.
	5	IG1: EMG BFB (5) baseline	IG1: EMG BFB (5) 8 weeks posttreatment		−9.30 [−12.61; −6.00]	−4.89 [H]	n.a.
	6	IG2: BFB skin temperature baseline (6)	IG2: BFB skin temperature posttreatment (6)		−2.00 [−8.86;4.86]	−0.44 [S]	n.a.
	6	IG2: BFB skin temperature baseline (6)	IG2: BFB skin temperature 8 weeks posttreatment (6)		−8.10 [−14.25; −1.95]	−2.00 [H]	n.a.
Bennink et al. 1982 [44]	5	IG1 (relaxation + EMG BFB) pretreatment (5)	IG1 (relaxation + EMG BFB) posttreatment (5)	Symptom score: SSS total of 15 symptoms (1 best −5 worst)	−3.6 [−14.27;7.07]	−0.59 [M]	n.a.
	5	IG1 (relaxation + EMG BFB) pretreatment (5)	IG1 (relaxation + EMG BFB) posttreatment (5)	SSS of subdomain cramps, backache, abdominal pain (1–5 = very severely)	−1.6 [−4.63;1.43]	−0.92 [L]	n.a.
	5	IG1 (relaxation + EMG BFB) pretreatment (5)	IG1 (relaxation + EMG BFB) posttreatment (5)	SSS of subdomain cramps only (1–5 = very severely)	−0.6 [−1.47;0.27]	−1.19 [L]	n.a.
Vagedes et al. 2019 [45]	20	IG1 (BFB group) pre-BFB (20)	IG1 (BFB group) post-BFB (20)	Mean NRS (0–10) pain during menstruation	−0.3 [−1.2/0.6]^g^	−0.2 [VS]^g^	n.a.
	37	IG1 (BFB group) postBFB (20)	No treatment CG (17)		0.9 [−2.10/0.30]^g^	−0.51 [M]^g^	*p* = 0.211
	43	IG1 (BFB group) postBFB (20)	IG2 (rhythmical massage) post treatment (23)		−0.6 [−1.82/0.40]^g^	−0.34 [S]^g^	*p* = 0.361
	20	IG1 (BFB group) pre-BFB (20)	IG1 (BFB group) post-BFB (20)	Maximum NRS (0–10) pain during menstruation	−0.5 [−1.4/0.3]^g^	−0.2 [S]^g^	n.a.
	37	IG1 (BFB group) post-BFB (20)	No-treatment CG (17)		0.6 [−2.18/0.74]^g^	−0.40 [S]^g^	*p* > 0.05
	43	IG1 (BFB group) post-BFB (20)	IG2 (rhythmical massage) post treatment (23)		−0.6 [−1.94/0.76]^g^	−0.23 [S]^g^	*p* > 0.05
Starr et al. 2013 [46]	694	Pre-BFB in pat. w urinary symptoms (694)	Post-BFB in pat. w urinary symptoms (n.a.)	% subjective global urinary symptom improvement since initial session (0: none–100%: perfect)	Mean 80–85% improvement^h^	n.a.	n.a.
	187	Pre-BFB in pat. w bowl symptoms (187)	Post-BFB in pat. w bowl symptoms (n.a.)	% subjective global bowel symptom improvement since initial session (0: none–100%: perfect)	Mean 80–85% improvement^h^	n.a.	n.a.
	368	Pre-BFB in pat. w pelvic pain symptoms (368)	Post-BFB in pat. w pelvic pain symptoms (n.a.)	% subjective global pelvic pain symptom improvement since the initial session (0: none–100%: perfect)	Mean 50–90% improve-ment^h^	n.a.	*p* > 0.05
Lúcio et al. 2014 [47]	6	IG1 pre BFB, PFM training and sham-electro-stimulation (6)	IG1 post BFB, PFM training and sham electrostimulation (6)	Symptom score: FSFI subdomain pain	1.6	n.a.	*p* > 0.05^b^
	6	IG1 pre BFB, PFM training and sham-electro-stimulation (6)	IG1 post BFB, PFM training and sham-electro-stimulation (6)	Symptom score: FSFI total score (2.0–36.0 = best)	−10	n.a.	***p*** **<** **0.05**^**b**^
Aalaie et al. 2020 [48]	9	IG1 (BFB group) pre-treatment (10)	IG1 (BFB group) 3 months post-treatment (9)	Symptom score: FSFI subdomain pain	0.9 [0.1;1.6]^g^	η2 = 0.66 [L]^g^	***p*** **=** **0.026**
	20	IG1 (BFB group) 3 months post-treatment (9)	IG2 (EGS) 3 months post-treatment (11)	–	n.a.	η2 = 0.01 [S]^g^	*p* = 0.985
	9	IG1 (BFB group) pre-treatment (10)	IG1 (BFB group) 3 months post-treatment (9)	Symptom score: FSFI total score (2.0–36.0 = best)	8.9 [7.0; 10.9]^g^	η2 = 0.96 [L]^g^	***p*** **<** **0.001**
	20	IG1 (BFB group) 3 months post-treatment (9)	IG2 (EGS) 3 months post-treatment (11)		n.a.	η2 = 0.64 [L]^g^	***p*** **=** **0.002**
**Chronic pelvic pain in children**							
Hoebeke et al. 2004 [51]	21	*n*/a	Study group post BFB (21)	SR: % patients reporting complete pain relief	80.95	n.a.	n.a.
	21	*n*/a	Study group long-term f/u (16 months) (21)		66.67	n.a.	n.a.
Ebiloglu et al. 2016 [52]	136	Whole study group pre-BFB [136]	Whole study group post BFB (6 months) (136)	SR: % patients with dysuria	−19.85	n.a.	***p*** **=** **0.007**^c^
	107	Subgroup OBS and dysf. voiding pre-BFB (107)	Post BFB (6 months) in this subgroup (107)		−20.56	n.a.	***p*** **<** **0.001**^c^
	29	Subgroup OBS only pre-BFB (29)	Post BFB (6 months) in this subgroup (29)		−17.24	n.a.	***p*** **<** **0.001**^c^
	136	Whole study group pre-BFB (136)	Whole study group post BFB (6 months) (136)	Symptom score: LUTDSS	−8.2	n.a.	***p*** **<** **0.001**^c^
Ergin et al. 2016 [53]	39	IG pat. w dysfunctional voiding pre-BFB in (52)	IG post BFB (6th month) (39)	SR: % patients with dysuria	−83.3	n.a.	*p* = 0.063^c^
	39	IG pat. w dysfunctional voiding pre-BFB (52)	IG post BFB (6th month) (39)	Symptom score: DVISSS	−8.3	n.a.	***p*** **=** **0.019**
Li et al. 2006 [54]	25	IG pat. w chronic prostatitis pre-BFB (25)	IG post BFB (after ~12 weeks) (25)	Symptom score: NIH-CPSI subdomain pain (0–21)	−2	n.a.	***p*** **=** **0.001**^**b**^
	25	IG pat. w chronic prostatitis pre-BFB (25)	IG post BFB (after ~12 weeks) (25)	Symptom score: NIH-CPSI total score (0–43)	−17	n.a.	***p*** **<** **0.001**^**b**^
**Musculoskeletal, low back pain, myofascial pain**							
Kent et al. 2015 [55]	58	IG pre-BFB (58)	IG 3 months post-BFB (58)	Pain VAS (0–10)	−20.5 [−30.45;−10.55]	−0.87 [VL]	n.a.
	54	IG pre-Guidelines Care (54)	IG 3 months post-Guidelines care (54)		−6.5 [−9.34;−3.61]	−0.98 [VL]	n.a.

effect sizes are Cohen’s d if not marked otherwise; criteria for determining effect sizes for Cohen’s d calculated by the authors: [VS]: very small effect size, [S]: small effect size, [M]: medium effect size, [L]: large effect size, [VL] very large effect size, [H] huge effect size; criteria for determining effect sizes for Cohen’s d calculated by the authors: [VS]: dz 0.01–< 0.20, [S]: dz < 0.5, [M]: dz < 0.8, [L]: dz < 1.2, [VL]: dz < 2.0, [H]: dz ≥ 2.0 according to [61, 63]

*AUA* symptom score: Americal Urological Association Symptom Score [59]; *BFB* biofeedback; *CG* control group; *CI* confidence interval; *DVISSS* Dysfunctional Voiding and Incontinence Symptom Scoring System (DVISSS) [64]; *EGS* electrogalvanic stimulation; *FSFI* Female Sexual Function Index total score [56]; *f/u* follow-up; *IG* intervention group; *IPPS* International Prostate Symptom Score [65]; *LAS* levator ani syndrome; *LUTDSS* Lower Urinary Tract Dysfunction Symptom Score [57]; *MD* mean difference, *n/a* not applicable; *n.a.* not available; *NIH-CPSI* National Institutes of Health Chronic Prostatitis Symptom Index [66]; *NMES* neuromuscular electrical stimulation; *NRS* Numeric Rating Scale [60]; *OBS* overactive bladder syndrome; *pat.* patient; *pat. w* patients with; *PEMF* pulsed electromagnetic field therapy; *PF* pelvic floor; *PFM* pelvic floor muscle; *QoL* quality of life; *SF-36* Short Form 36 [67]; *SR* success rate; *SSS* Symptom Severity Score [58]; *UCPPS* Urological Chronic Pelvic Pain Syndrome, *VAS* Visual Analog Scale [60]; *w* with

^a^t‑test, ^b^Wilcoxon, ^c^McNemar, ^d^ANOVA, ^e^ANCOVA; ^f^χ^2^, %: percent, ^g^effect sizes and confidence intervals stated by authors of original studies (not marked: values calculated by review authors), ^h^Inconsistent charting in source data according to study authors

**Table 6:** Primary outcome: effect of biofeedback interventions on quality of life

Study	*n* (total group 1, 2)	Group 1 (n1)	Group 2 (n2)	Outcome measure	MD: group 2 minus group 1	95% CI lower bound	95% CI upper bound	Effect size [strength]		*P*-value (for difference in means)
**Constipation, dyssynergic defecation**										
Ba-Bai-Ke-Re et al. 2014 [26]	88	IG1 (BFB) (44)	IG2 (laxatives) (44)	PAC-QoL 1 month post	12.00	10.904	13.096	3.731	H	***p*** **<** **0.001**^**a**^
	88	IG1 (BFB) (44)	IG2 (laxatives) (44)	PAC-QoL 3 months post	14.00	13.077	14.923	5.173	H	***p*** **<** **0.001**^**a**^
	88	IG1 (BFB) (44)	IG2 (laxatives) (44)	PAC-QoL 6 months post	16.00	15.299	16.701	7.784	H	***p*** **<** **0.001**^**a**^
Zhu et al. 2011 [29]	36	Study group pre-BFB (36)	Study group post-BFB (36)	SF-36: physical functioning	7.30	−0.679	15.279	0.494	S	***p*** **=** **0.001**^**a**^
	36	Pre-BFB (36)	Post-BFB (36)	SF-36: role physical	23.80	2.347	45.253	0.599	M	***p*** **<** **0.001**^**a**^
	36	Pre-BFB (36)	Post-BFB (36)	SF-36: bodily pain	10.30	−1.301	21.901	0.479	S	***p*** **=** **0.001**^**a**^
	36	Pre-BFB (36)	Post-BFB (36)	SF-36: vitality	8.00	−3.932	19.932	0.362	S	***p*** **=** **0.042**^**a**^
	36	Pre-BFB (36)	Post-BFB (36)	SF-36: role emotional	19.50	0.387	38.613	0.551	M	***p*** **=** **0.001**^**a**^
	36	Pre-BFB (36)	Post-BFB (36)	SF-36: mental health	11.00	0.368	21.632	0.559	M	***p*** **=** **0.003**^**a**^
	36	Pre-BFB (36)	Post-BFB (36)	SF-36: social function	10.90	−0.701	22.510	0.507	M	***p*** **=** **0.014**^**a**^
	36	Pre-BFB (36)	Post-BFB (36)	SF-36: general health	10.50	−1.705	22.704	0.465	S	***p*** **=** **0.008**^**a**^
	36	Pre-BFB (36)	Post-BFB (36)	PAC-QOL: physical discomfort	−0.99	−1.561	−0.419	−0.937	L	***p*** **<** **0.001**^**a**^
	36	Pre-BFB (36)	Post-BFB (36)	PAC-QOL: psycho-social discomfort	−0.37	−0.784	0.044	−0.482	S	***p*** **<** **0.001**^**a**^
	36	Pre-BFB (36)	Post-BFB (36)	PAC-QOL: worries, concerns	−0.98	−1.420	−0.540	−1.201	VL	***p*** **<** **0.001**^**a**^
	36	Pre-BFB (36)	Post-BFB (36)	PAC-QOL: satisfaction	−1.33	−1.834	−0.826	−1.425	VL	***p*** **<** **0.001**^**a**^
	36	Pre-BFB (36)	Post-BFB (36)	PAC-QOL: overall	−0.92	−1.277	−0.563	−1.393	VL	***p*** **<** **0.001**^**a**^
**Male chronic pelvic pain syndrome, Urological Chronic Pelvic Pain Syndrome**										
Cornel et al. 2005 [34]	31	Study group pre-BFB (31)	Study group post-BFB (31)	NIH-CPSI: QoL (0–12 points)	−3.80	n.a.	n.a.	n.a.	n.a.	***p*** **<** **0.001**^**b**^
Yang et al. 2017 [35]	22	IG1 (BFB + NMES) pretreatment (22)	IG1 12 weeks post-treatment (22)	NIH-CPSI: QoL	−5.20	−7.523	−2.870	−1.564	VL	***p*** **<** **0.001**^**a**^
	45	BFB + NMES (22)	PEMF (23)	NIH-CPSI: QoL 12 wks post	1.20	−0.382	2.782	0.365	S	***p*** **=** **0.012**^**a**^
He et al. 2009 [36]	21	Study group pre-BFB (21)	Study group 10 weeks post-BFB (21)	NIH-CPSI: life impact	−6.70	−8.605	−4.795	−2.528	H	***p*** **<** **0.05**^**b**^
**Female chronic pelvic pain**										
Gentilcore-Saulnier et al. 2010 [40]	11	IG (provoked vestibulodynia) pre-BFB (11)	IG post-BFB (11)	Perceived impact on QoL (0 = no to 10 = worst)	−1.55	−3.367	0.267	−0.882	L	***p*** **=** **0.003**^**a**^
Bendana et al. 2009 [41]	13	Strudy group pre-BFB (52)	Study group 3 months post-BFB (13)	VAS (0–10)	−2.56	n.a.	n.a.	n.a.	n.a.	***p*** **<** **0.001**^**a**^
Vagedes et al. 2019 [45]	20	IG1 (BFB) pre-BFB (20)	IG1 post-BFB (20)	SF-12: mental score	4.1^c^	−0.3^c^	8.4^c^	0.4^c^	S	n.a.
	20	IG1 (BFB) pre-BFB (20)	IG1 post-BFB (20)	SF-12: physical score	4.4^c^	0.4^c^	8.5^c^	0.5^c^	S	n.a.
	20	IG1 (BFB) pre-BFB (20)	IG1 post-BFB (20)	SF-12: sum score	8.7^c^	3.5^c^	13.8^c^	0.6^c^	M	n.a.
	37	IG1 (BFB) post-BFB (20)	CG (usual care) (17)	SF-12: sum score	6.13^c^	−3.09^c^	15.35^c^	0.41^c^	S	*p* > 0.05
	43	IG1 (BFB) post-BFB (20)	IG2 (massage) post-treatment (23)	SF-12: sum score	−0.57^c^	−9.18^c^	8.03^c^	−0.04^c^	VS	*p* > 0.05
**Chronic pelvic pain in children**										
Li et al. 2006 [54]	22	IG patients with chronic prostatitis post-BFB (25)	IG post-BFB (22)	NIH-CPSI: life impact	8	n.a.	n.a.	n.a.	n.a.	***p*** **<** **0.001**^**b**^

effect size strength: [VS]: very small effect size, [S]: small effect size, [M]: medium effect size, [L]: large effect size, [VL] very large effect size, [H] huge effect size; criteria for determining effect sizes for Cohen’s *d *calculated by the authors (^c^): [VS]: *dz *0.01–<0.20, [S]: *dz* < 0.5, [M]: *dz* < 0.8, [L]: *dz* < 1.2, [VL]: *dz* < 2.0, [H]: *dz* ≥ 2.0 according to [61, 63]; criteria for determining effect sizes not calculated by the authors are stated in the respective studies; effect sizes and confidence intervals which were calculated by the review authors are not marked, those effect sizes and confidence intervals that are stated in respective paper are marked with (^c^)

*BFB* biofeedback, *CG* control group, *CI* confidence interval, *IG* intervention group, *MD* mean difference, *n.a.* data not available, *NIH-CPSI* National Institutes of Health Chronic Prostatitis Symptom Index [66], *NMES* neuromuscular electrical stimulation, *PEMF* pulsed electromagnetic field therapy, *QoL* quality of life, *SF-12* Short Form-12 [68], *SF-36* Short Form 36 [67], *UCPPS* Urological Chronic Pelvic Pain Syndrome, *VAS* visual analog scale; *wk/wks* week(s)

^a^t‑test, ^b^Wilcoxon

**Table 7:** Secondary outcome: Effect of biofeedback interventions on physiological parameters

Study	Secondarily evaluated outcome measure	Domain, subgroup	f/u	IG pre-post	IG vs. IG /IG vs. CG	Significant improvement in subdomains, significant difference between IG/IG or IG/CG (*p*-value)	No significant improvement in subdomains or no significant difference between IG/IG, IG/CG (*p*-value)
**Anorectal pain syndrome**							
Chiarioni et al. 2010 [16]	Anorectal manometry	IG1 BFB group: patients with high likely LAS	Baseline–1 month	x	–	Anal pressure with straining (% relaxing), balloon defecation (% successful), urge threshold (ml), maximum tolerable volume (ml), compliance (mm Hg) (*p* < 0.025)	Resting anal canal pressure (mm Hg), rectoanal inhibitory reflex threshold (ml) (*p* ≥ 0.025)
			Baseline–3 month	x	–	Anal pressure with straining (% relaxing), balloon defecation (% successful), rectoanal inhibitory reflex threshold (ml), urge threshold (ml), maximum tolerable volume (ml) (*p* < 0.025)	Resting anal canal pressure (mm Hg), compliance (mm Hg) (*p* ≥ 0.025)
		IG1 BFB group: patients with possible LAS	Baseline—1 month, baseline—3 months	x	–	Anal pressure with straining (% relaxing), balloon defecation (% successful) (*p* < 0.025)	Resting anal canal pressure (mm Hg), rectoanal inhibitory reflex threshold (ml), urge threshold (ml), maximum tolerable volume (ml), compliance (mm Hg) (*p* < 0.025)
		IG1 BFB group (*n* = 52) vs. IG2 EGS (*n* = 52) or IG3 massage group (*n* = 53) (in favour of BFB) in patients with high likely LAS	After 1 month, after 3 months	–	x	Anal pressure with straining (% relaxing), balloon defecation (% successful) (*p* < 0.025)	Resting anal canal pressure (mm Hg), rectoanal inhibitory reflex threshold (ml), urge threshold (ml), maximum tolerable volume (ml), compliance (mm Hg) (*p* < 0.025)
Heah et al. 1997 [17]	Anorectal manometry	Study group (*n* = 16)	Baseline—after treatment	x	–	None (*p* < 0.05^2^)	Anal canal mean resting/maximum squeeze pressure (mm Hg), rectum volume first sensation (ml)/maximum tolerable volume (ml)/compliance (ml/mm Hg), perineal descent rest/strain (cm) (*p* > 0.05)
Grimaud et al. 1991 [20]	Anorectal manometry	Study group (*n* = 12)	Baseline—after treatment	x	–	Anal canal resting pressure (mm Hg), *p* < 0.01 (no significant difference any more compared to healthy controls without BFB intervention)	–
**Constipation, dyssynergic defecation**							
Chiarioni et al. 2006 [21]	Anorectal manometry	BFB group (*n* = 54/109)	Baseline—6 months, baseline—12 months after starting treatment	x	–	Increased anal pressure (*n*, %), (paradoxical) EMG increase (*n*, %), unable to evacuate balloon (*n*, %), anal squeeze pressure (mm Hg), rectoanal inhibitory reflex threshold (ml), urge threshold (ml), maximum tolerable volume (ml), compliance (mm Hg) (*p* < 0.01)	Anal resting pressure (mm Hg) (*p* ≥ 0.01)
			Baseline—24 months after starting treatment	x	–	Increased anal pressure (*n*, %), (paradoxical) EMG increase (*n*, %), unable to evacuate balloon (*n*, %), anal squeeze pressure (mm Hg), urge threshold (ml), maximum tolerable volume (ml) (*p* < 0.01)	Anal resting pressure (mm Hg), rectoanal inhibitory reflex threshold (ml), compliance (mm Hg) (*p* ≥ 0.01)
		BFB group vs. laxative group, in favour BFB group (*n* = 109)	6 and after 12 months after starting treatment	–	x	Increased anal pressure (*n*, %), (paradoxical) EMG increase (*n*, %), unable to evacuate balloon (*n*, %) (*p* < 0.01)	Anal resting pressure (mm Hg), anal squeeze pressure (mm Hg), rectoanal inhibitory reflex threshold (ml), urge threshold (ml), maximum tolerable volume (ml), compliance (mm Hg)
			24 months after starting treatment	–	x	No parameter (*p* < 0.01)	All parameters (*p* ≥ 0.01)
Koutsomanis et al. 1994 [22]	Anorectal manometry	Study group (*n* = 20)	Baseline—after treatment	x	–	Paradoxical contraction on evacuation straining (*n* pre: *n* = 15/20, *n* post: *n* = 0/20, (*p* : n.a.))	Anal resting pressure, anal squeeze pressure, anorectal sensation (*p* : n.a.)
Battaglia et al. 2004 [24]	Anorectal manometry	Patients with PF dyssynergia (*n* = 14/24)	Baseline—3 months after treatment	x	–	Sensation threshold (mm Hg; *p* = 0.042), paradoxical increase in intra-anal pressure during straining (*p* : n.a.)	Maximum basal pressure of internal anal sphincter, maximum rectum tolerable volume (*p* ≥ 0.05)
		Patients with slow transit (*n* = 10/24)	Baseline—3 months after treatment	x	–	Maximum rectum tolerable volume (ml), (*p* = 0.008)	Maximum basal pressure of internal anal sphincter (mm Hg), sensation threshold (mm Hg) (*p* ≥ 0.05)
Wang et al. 2003 [25]	Anorectal manometry	Study group (*n* = 50)	Baseline—after treatment	x	–	Anal canal average rest pressure (mm Hg) rectum: initial sense (ml), (*p* < 0.05)	Anal canal voluntary squeeze (mm Hg), rectum: maximum tolerable volume (ml) and compliance (ml/mm Hg), (*p* ≥ 0.05)
Ba-Bai-Ke-Re et al. 2014 [26]	Anorectal manometry	BFB group vs. laxative group, in favor BFB group (*n* = 88)	Baseline—after treatment	–	x	Anorectal resting pressure, anorectal squeeze pressure (mm Hg) (*p* < 0.05)	–
Chiarioni et al. 2005 [28]	Gut transit time	PF dyssynergia (*n* = 34) vs. slow transit only (*n* = 12)	Baseline—1/6/12/24 months after treatment	–	x	% of patients with abnormal transit test: baseline: 100%; at all f/u: PF dyssynergia vs. slow-transit-only: sign. smaller % of patients with abnormally delayed transit, *p* < 0.05	–
	Balloon defecation test	Patients with PF dyssynergia (*n* = 34)	Baseline—after treatment (1, 6, 12, 24 months)	x	–	Baseline: 0%, after treatment (1–24 months): 82–85% could defecate the balloon within 5 min (*p* : n.a.)	–
	Anorectal manometry	Patients with PF dyssynergia (*n* = 34)	Baseline—after treatment (1, 6 months)	x	–	Urge threshold (ml), maximum tolerable pressure (mm Hg), straining rectal pressure (mm Hg), dyssynergia (balloon defecation test) (*p* < 0.05)	Anal canal resting pressure (ml), rectoanal inhibitory reflex threshold (ml), compliance (mm Hg100ml) *p* ≥ 0.05
		Patients with slow transit only (*n* = 12)	Baseline—after treatment (1, 6 months)	x	–	Urge threshold (ml) (*p* < 0.05)	Anal canal resting pressure (ml), rectoanal inhibitory reflex threshold (ml), maximum tolerable pressure (mm Hg), compliance (mm Hg100ml), straining rectal pressure (mm Hg) (*p* ≥ 0.05)
		Patients with PF dyssynergia (*n* = 34) vs. slow transit only (*n* = 12) in favour of PF dyssynergia	After 1, 6 months	–	x	Rectoanal inhibitory reflex threshold (ml; only after 1 month), after 1, 6 months: urge threshold (ml), maximum tolerable pressure (mm Hg), *p* < 0.01	Rectoanal inhibitory reflex threshold (ml, only after 6 months), after 1, 6 months: anal canal resting pressure (ml), rectoanal inhibitory reflex threshold (ml, only after 6 months), compliance (mm Hg100ml), straining rectal pressure (mm Hg) (*p* ≥ 0.01)
Parker et al. 2019 [31]	Anorectal manometry	Whole study group (constipation, fecal incontinence, rectal pain) (*n* = 130)	Baseline—after treatment	x	–	Resolved dyssynergic manometric pressure profile, balloon expulsion test < 1 min in *n* = 27/130, (*p* : n.a.)	–
		Constipation + dyssynergic defecation (*n* = 33/130)	Baseline—after treatment	x	–	Resolved dyssynergic manometric pressure profile, balloon expulsion test < 1 min in *n* = 13/53, (*p* : n.a.)	–
		Constipation without dyssynergic defecation (*n* = 3/130)	Baseline—after treatment	x	–	Improvement in anorectal manometry profile (resolved dyssynergic manometric pressure profile, balloon expulsion test < 1 min) in *n* = 2/3, (*p* : n.a.)	–
		Rectal pain (*n* = 3/130)	Baseline—after treatment	x	–	Resolved dyssynergic manometric pressure profile, balioon expulsion test < 1 min in *n* = 2/3, (*p* : n.a.)	–
**Male chronic pelvic pain syndrome, Urological Chronic Pelvic Pain Syndrome**							
Cornel et al. 2005 [34]	Levator ani EMG	Study group (*n* = 18)	Baseline—after treatment	x	–	Mean pelvic muscle tonus↓ (mcV, *p* < 0.001^2^)	–
He et al. 2010 [36]	Urodynamics (uroflowmetry + EMG)	Study group (*n* = 21)	Baseline—10 wks after treatment	x	–	Max. flow rate (ml/s), max. detrusor pressure-storage phase (cmH2O), max. urethra closure pressure (cmH2O), max. urethral pressure (cmH2O) (*p* < 0.05)	–
**Female chronic pelvic pain**							
Glazer et al. 1995 [38]	PF EMG	Study group (*n* = 33)	Baseline—after treatment (=after 6th assessment)	x	–	Muscle contractile strength = mean contraction amplitude (mcV) ↑, mean relaxation amplitude ↓ (mcV) (*p* < 0.0001), SD: measure of the stability of the muscle at rest improved (*p* : n.a.)	–
McKay et al. 2001 [39]	PF EMG	Study group (*n* = 29)	After 1 month—after 6 months	x	–	Mean maximum contractile strength ↑ (mcV): after 1^st^ month: 16.42 in *n* = 29, after 6^th^ month: 42.73 in *n* = 11 (dropout *n* = 18), (*p* : n.a.)	–
Gentilcore-Saulnier et al. 2010 [40]	PF EMG	IG (provoked vestibulodynia, *n* = 11)	Baseline—after treatment	x	–	–	Tonic surface EMG resting activity: deep (*p* = 0.86) or superficial (*p* = 0.32) PF muscle layer
			Baseline—after treatment	x	–	–	PF muscle maximum voluntary contractile activity: deep: *p* = 0.82; superficial: *p* = 0.50
			Baseline—after treatment	x	–	Superficial PFM EMG activity pain responses ↓ (mcV), (*p* < 0.0001)	Deep PF layer EMG activity pain response (mcV), (*p* = 0.72)
		IG (provoked vestibulodynia, *n* = 11) vs. healthy CG (*n* = 11)	Baseline, pretreatment	–	x	Sign. greater superficial PFM EMG activity pain responses (mcV) in pretreatment IG compared to CG (*p* = 0.003); sign. higher tonic activity in superficial PFM in pretreatment IG compared to CG (*p* = 0.04)	No sign. difference pretreatment IG vs. CG for: PFM maximum voluntary contractile activity for deep (*p* = 0.81) and superficial (*p* = 0.36) PFM; EMG pain responses of the deep PFM (*p* = 0.89); deep PFM tonic activity (*p* = 0.18)
		IG (provoked vestibulodynia, *n* = 11) vs. healthy CG (*n* = 11)	After treatment	–	x	–	No sign. difference between posttreatment IG and CG: tonic surface EMG resting activity at both superficial (*p* = 0.82) and deep (*p* = 0.31) PFM; PFM maximum voluntary contractile activity for deep (*p* = 0.54) and superficial (*p* = 0.90) PFM; EMG activity pain response (mcV) for deep (*p* = 0.98) or superficial (*p* = 0.18) PFM
	Digital intravaginal assessment	IG (*n* = 11 with pelvic floor dyssynergia)	Baseline—after treatment	x	–	PFM tone ↓ (*p* < 0.001), PFM flexibility ↑ (*p* = 0.01), PFM post-contraction relaxation capacity ↑ (*p* = 0.05), PFM strength ↑ (*p* = 0.04)	–
		IG (*n* = 11 with provoked vestibulodynia) vs. healthy CG (*n* = 11)	Baseline	–	x	Pretreatment PVD group vs. CG: PFM tone: sign. higher in PVD group (*p* = 0.005), PFM flexibility: sign. lower in PVD group (*p* = 0.01), PFM relaxation: sign. less ability to relax PFM in PVD group (*p* = 0.02) compared to CG	Pretreatment PVD group vs. CG: no significant difference in PFM strength (*p* = 0.54)
		IG (*n* = 11 patients with provoked vestibulodynia) vs. healthy CG (*n* = 11)	After treatment	–	x	–	posttreatment PVD group vs. CG: no sign. difference in PFM tone (*p* = 0.30), PFM flexibility (*p* = 1.00), PFM relaxation (*p* = 0.47), PFM strength (*p* = 0.12)
Philips et al. 1992 [42]	Perivaginal EMG	IG 1 (BFB group, *n* = 10)	Baseline—after treatment	x	–	–	Mean EMG scores (seated to void, tensing, relaxing, voiding; mcV) *p* > 0.05
Bennink et al. 1982 [44]	EMG lower abdomen	IG1 (*n* = 5) vs. IG2 (*n* = 5)	–	–	x	BFB group maintained a significantly lower level of EMG muscle tone (mcV/s) of lower abdomen on 1^st^ day of menstruation compared to massage group (*p* < 0.05)	–
Vagedes et al. 2019 [45]	Heart rate variability	BFB group (*n* = 20)	Baseline—after treatment	x	–	–	SDNNl, RMSSD, LF/HF ratio (*p* > 0.05)
		BFB (*n* = 20) vs. CG (*n* = 17)/BFB vs. massage (*n* = 23)	After treatment	–	x	–	Same values: BFB vs. CG/massage vs. BFB group post treatment: *p* > 0.05
Lúcio et al. 2014 [47]	Intravaginal digital examination	IG1: EMG BFB + PF training + sham NMES (*n* = 6)	Baseline—after treatment	x	–	PF muscle function according to PERFECT scheme ↑ [69]: power (0–5 = max. strength), endurance (sec), dynamic endurance (no. of repetitions), fast contractions (no. of repetitions): *p* < 0.05	PF muscle palpation score: PF muscle tone (score: −3 to +3 = very hypertonic), flexibility (score 0–4 = very flexible), ability to relax PF muscles (Score 0–4 = spastic): *p* > 0.05
**Chronic pelvic pain in children**							
Ebiloglu et al. 2016 [52]	Urodynamics	Study group (*n* = 136)	Baseline—after treatment (6 months)	x	–	No. of patients with positive perineal EMG activity while urinating (*p* < 0.001)	–
	Urodynamics	Study group (*n* = 136)	Baseline—after treatment (6 months)	x	–	Mean voided volume (ml, *p* = 0.019), mean maximum flow rate (ml/s, *p* = 0.012)	Mean average flow rate (ml/s, *p* = 0.209), mean voiding time (s, *p* = 0.345), post-void residual volume (ml, *p* = 0.374)
Ergin et al. 2016 [53]	Urodynamics	Intervention group (*n* = 39)	Baseline—after treatment (6 months)	x	–	Uroflowmetry—EMG, post-void residual volume (*p* < 0.001)	–
Li et al. 2006 [54]	Urodynamics	IG (*n* = 25)	Baseline—after treatment	x	–	Maximum urinary flow rate (ml/s), *p* = 0.001	Postvoid residual urine volume (ml), *p* = 0.08

*BFB* biofeedback, *CG* control group (no intervention), *EGS* electrogalvanic stimulation, *EMG* electromyography, *f/u* follow-up, *IG* intervention group, *LAS* levator ani syndrome, LF/HF ratio  ratio of two bands from frequency domain analysis: LF band (0.04–0.15 Hz) indicating sympathetic and parasympathetic activity, HF band (0.15–0.40 Hz) indicating parasympathetic activity; *mcV* microvolt, *ml* mililiter, *ml/* mililiter per second, *no*. number(s), *PF* pelvic floor, *PFM* pelvic floor muscle(s), *RMSSD* root mean square of successive differences; *s* second(s), *SD* standard deviation, *SDNN* standard deviation of normal to normal, *vs*. versus, *wk, wks* week(s)

#### Data synthesis

An attempt was made to bundle data for a meta-analysis; however, due to the substantial heterogeneity of study designs, patient characteristics, interventions and effect measures, a meta-analysis was not possible as results are considered unreliable when a small number of heterogeneous studies are assessed [70]. Rather, a narrative synthesis of study results was performed [71], and findings were juxtaposed in the respective tables to provide a comprehensive overview of the current literature.

#### Quality assessment

As trials differed in their study design, the McMaster Critical Review Form—Quantitative Studies [72, 73] was chosen for assessing the methodological quality of all studies included. This critical appraisal tool allows comparisons across different types of quantitative study designs due to its generic composition [5, 74, 75]. It comprises 15 items that evaluate method rigor and bias and has a guideline for completing the questionnaire that facilitates consistency in interpretation and application [72, 74]. In its original form, the tool did not provide a numerical summation. Based on previous reviews [5, 74, 75], for better comparability between included studies, a sum score of the respective subdomains was established. Each question is rated with either “yes” (1 point), “no”, “not addressed” or “not applicable (*N*/A)” (0 points). In this arbitrary scoring system, higher scores indicate higher methodological quality, resulting in a possible total score of 14 points [5].

In addition, studies with an RCT design were evaluated using the Physiotherapy Evidence Database—PEDro score, a valid and reliable tool for assessing the methodological quality and completeness of statistical reporting of randomized and quasi randomized controlled trials in physiotherapy [12, 76–81]. The tool evaluates internal validity and interpretability [82]. Eleven items are rated yes or no (1 or 0 points) according to whether the criterion is clearly satisfied in the study. A total PEDro score is achieved by adding the ratings of items 2–11 for a total score between 0 and 10. Higher scores indicate superior methodological quality. Studies with 9–10 points are considered excellent, 6–8 good, 4–5 fair and < 4 poor quality [80].

## Results

### Study selection

**Fig. 1:**
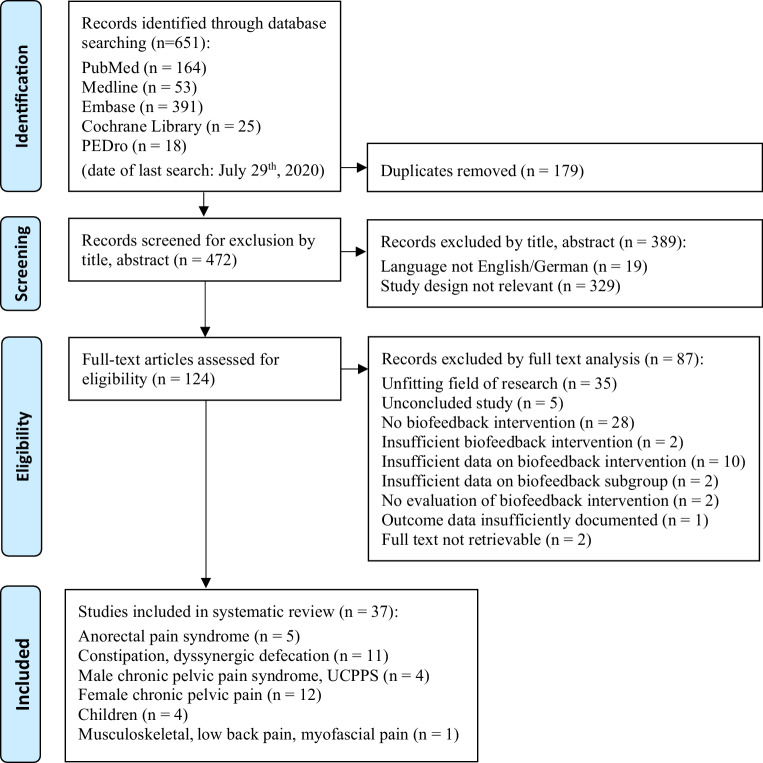
Flow chart of the systematic literature search and the selection according to the Preferred Reporting Items for Systematic Reviews and Meta-Analyses (PRISMA) guidelines. *UCPPS* urological chronic pelvic pain syndrome

A total of 651 studies published between 1978 and 29 July 2020 were found and screened for eligibility by title and abstract. After eliminating duplicates, 389 studies were rejected as non-includable, 83 studies were selected for full-text analysis and 37 articles corresponded to the inclusion criteria. Details on the systematic literature search and the selection process are presented in Fig. 1.

### Study characteristics

#### Quality assessment

Table 8 shows the quality assessment using the Mc Master Critical Review Form—Quantitative Studies Tool for assessing the risk of bias of all studies included.

**Table 8:** Methodological quality assessment: evaluating all studies included (*n* = 37): McMaster Critical Review Form (CRF)—Quantitative Studies [72]

Study	Study design	Was the purpose stated clearly?	Was relevant background literature reviewed?	Was the sample described in detail?	Was sample size justified?	Were the outcome measures reliable?	Were the outcome measures valid?	Intervention was described in detail?	Contamination was avoided?	Cointervention^a^ was avoided?	Results were reported in terms of statistical significance?	Were the analysis method(s) appropriate?	Clinical importance was reported?	Drop-outs were reported?	Conclusions were appropriate given study methods and results?	Total score (sum score)
**Anorectal pain syndrome**																
Chiarioni et al. 2010 [16]	RCT, 3 arms	Yes	Yes	Yes	Yes	*N.add.*^a^	*N.add.*^a^	Yes	Yes	No	Yes	Yes	Yes	No	Yes	10
Heah et al. 1997 [17]	Non-RCT	Yes	Yes	No	No	*N.add.*^a^	*N.add.*^a^	Yes	*N*/A	No	Yes	Yes	Yes	No	Yes	7
Ger et al. 1993 [18]	Non-RCT	Yes	Yes	Yes	No	*N.add.*	*N.add.*	Yes	No	*N.add.*	No	No	Yes	Yes	Yes	7
Gilliland et al. 1997a [19]	Non-RCT, retrospective	Yes	Yes	Yes	No	*N.add.*	*N.add.*	No	*N*/A	*N.add.*	Yes	Yes	Yes	Yes	Yes	8
Grimaud et al. 1991 [20]	Non-RCT	Yes	Yes	Yes	No	*N.add.*^a^	*N.add.*^a^	Yes	Yes	Yes	Yes	Yes	Yes	Yes	Yes	11
**Constipation**																
Chiarioni et al. 2006 [21]	RCT, 2 arms	Yes	Yes	Yes	Yes	Yes	Yes	Yes	Yes	No	Yes	Yes	Yes	Yes	Yes	13
Koutsomanis et al. 1994 [22]	Non-RCT	Yes	Yes	No	No	*N.add.*^a^	*N.add.*^a^	Yes	*N*/A	No	Yes	Yes	Yes	Yes	No	7
Chiotakakou-Faliakou et al. 1998 [23]	Non-RCT, retrospective	Yes	Yes	Yes	No	*N.add.*	*N.add.*	Yes	*N*/A	No	Yes	Yes	Yes	Yes	Yes	9
Battaglia et al. 2004 [24]	Non-RCT	Yes	Yes	Yes	No	*N.add.*^a^	*N.add.*^a^	Yes	*N*/A	No	Yes	Yes	Yes	No	Yes	8
Wang et al. 2003 [25]	Non-RCT	Yes	Yes	Yes	No	*N.add.*^a^	Yes	Yes	Yes	No	Yes	Yes	Yes	No	Yes	10
Ba-Bai-Ke-Re et al. 2014 [26]	RCT, 2 arms	Yes	Yes	no	Yes	*N.add.*^a^	Yes	Yes	Yes	No	Yes	Yes	Yes	Yes	Yes	11
Roy 2000 [27]	Non-RCT, retrospective	Yes	Yes	Yes	No	*N.add.*	*N.add.*	Yes	*N*/A	No	Yes	Yes	Yes	No	Yes	8
Chiarioni et al. 2005 [28]	Non-RCT	Yes	Yes	Yes	No	*N.add.*^a^	*N.add.*^a^	Yes	*N*/A	No	Yes	Yes	Yes	Yes	Yes	9
Zhu et al. 2011 [29]	Non-RCT	Yes	Yes	Yes	Yes	Yes	Yes	Yes	*N*/A	No	Yes	Yes	Yes	Yes	No	11
Gilliland et al. 1997b [30]	Non-RCT, retrospective	Yes	Yes	Yes	No	*N.add.*	*N.add.*	No	*N*/A	No	Yes	Yes	Yes	Yes	No	7
Parker et al. 2019 [31]	Non-RCT, retrospective	Yes	Yes	No	No	*N.add.*	No	No	*N*/A	*N.add.*	No	No	Yes	Yes	Yes	5
**Male chronic pelvic pain syndrome, Urological Pelvic Pain Syndrome**																
Clemens et al. 2000 [32]	Non-RCT	Yes	Yes	No	No	*N.add.*	*N.add.*	Yes	*N*/A	*N.add.*	Yes	Yes	Yes	Yes	Yes	8
Cornel et al. 2005 [34]	Non-RCT	Yes	Yes	Yes	No	Yes	Yes	Yes	*N*/A	*N.add.*	Yes	Yes	Yes	Yes	Yes	11
Yang et al. 2017 [35]	Non-RCT, retrospective	Yes	Yes	Yes	No	*N.add.*^a^	Yes	Yes	Yes	Yes	Yes	Yes	Yes	Yes	Yes	12
He et al. 2010 [36]	Non-RCT, retrospective	Yes	No	No	No	*N.add.*^a^	*N.add.*^a^	No	*N*/A	Yes	Yes	Yes	Yes	No	Yes	6
**Female chronic pelvic pain**																
Schmitt et al. 2017 [37]	Non-RCT	Yes	Yes	No	No	*N.add.*^a^	Yes	Yes	*N*/A	*N.add.*	Yes	Yes	Yes	Yes	Yes	9
Glazer et al. 1995 [38]	Non-RCT	No	Yes	Yes	No	*N.add.*^a^	*N.add.*^a^	Yes	*N*/A	No	Yes	Yes	Yes	No	Yes	7
McKay et al. 2001 [39]	Non-RCT	Yes	Yes	Yes	No	*N.add.*^a^	*N.add.*^a^	Yes	*N*/A	*N.add.*	Yes	Yes	Yes	Yes	Yes	9
Gentilcore-Saulnier et al. 2010 [40]	Non-RCT	Yes	Yes	Yes	Yes	Yes	Yes	Yes	*N*/A	Yes	Yes	Yes	Yes	No	Yes	12
Bendana et al. 2009 [41]	Non-RCT, retrospective	Yes	Yes	Yes	No	*N.add.*	No	Yes	*N*/A	*N.add.*	Yes	Yes	Yes	Yes	Yes	9
Philips 1992 [42]	RCT	Yes	Yes	Yes	No	*N.add.*^a^	*N.add.*^a^	No	Yes	*N.add.*	Yes	Yes	Yes	No	Yes	8
Hart et al. 1981 [43]	Non-RCT, 2 arms	Yes	Yes	Yes	No	*N.add.*	*N.add.*	Yes	Yes	*N.add.*	Yes	*N.add.*	Yes	Yes	No	8
Bennink 1982 [44]	RCT, 3 arms	Yes	Yes	No	No	*N.add.*	*N.add.*	Yes	Yes	Yes	Yes	No	Yes	Yes	Yes	9
Vagedes et al. 2019 [45]	RCT, 3 arm	Yes	Yes	Yes	Yes	Yes	*N.add.*^a^	Yes	Yes	No	Yes	Yes	Yes	Yes	Yes	12
Starr et al. 2013 [46]	Non-RCT, retrospective	Yes	Yes	Yes	Yes	*N.add.*	No	Yes	*N*/A	No	Yes	Yes	Yes	Yes	Yes	10
Lúcio et al. 2014 [47]	RCT, 3 arms	Yes	Yes	Yes	No	*N.add.*^a^	Yes	Yes	Yes	*N.add.*	Yes	Yes	Yes	Yes	Yes	11
Aalaie et al. 2020 [48]	RCT, 2 arms	Yes	Yes	Yes	Yes	Yes	*N.add.*^a^	Yes	Yes	*N.add.*	Yes	Yes	Yes	Yes	Yes	12
**Chronic pelvic pain in children in children**																
Hoebeke et al. 2004 [51]	Non-RCT	Yes	Yes	Yes	No	*N.add.*	*N.add.*	Yes	*N*/A	No	No	*N.add.*	Yes	No	No	5
Ebiloglu et al. 2016 [52]	Non-RCT, retrospective	Yes	Yes	Yes	Yes	*N.add.*^a^	*N.add.*^a^	Yes	*N*/A	*N.add.*	Yes	Yes	Yes	No	Yes	9
Ergin et al. 2016 [53]	Non-RCT	Yes	Yes	Yes	No	*N.add.*^a^	Yes	No	*N*/A	*N.add.*	Yes	Yes	Yes	Yes	Yes	9
Li et al. 2006 [54]	Non-RCT	Yes	Yes	Yes	No	*N.add.*^a^	Yes	No	*N*/A	Yes	Yes	Yes	Yes	Yes	Yes	10
**Musculoskeletal, low back pain, myofascial pain**																
Kent et al. 2015 [55]	RCT, 2 arms	Yes	Yes	Yes	Yes	Yes	Yes	Yes	Yes	No	Yes	Yes	Yes	Yes	Yes	13

McMaster CRF: 15 items; total score = 14; study design item does not contribute to total score; codes: yes = 1, no = 0, *N/A* (not applicable) = 0, *N.add* not addressed (no information provided in the study) = 0 [5]; ^a^not addressed in respective paper, but at least one main outcome tool was judged to be valid/reliable by review authors (described in the literature)

Total score: higher scores indicate higher methodological quality, resulting in a possible total score of 14 points.

All studies but one were judged to have clearly stated the purpose of the study [38] and to have reviewed the relevant background literature [36]. The majority of the studies (29/37) gave enough detail on important sample characteristics. Only 10/37 studies stated how they arrived at the sample size. A minority of studies explicitly stated to have used reliable [21, 29, 34, 40, 45, 48, 55] and valid [21, 25, 26, 34, 35, 37, 40, 47, 53–55] outcome measures. For several tools, however, the psychometric properties are described in the literature. If at least one main outcome tool was used that is described in the literature, studies were marked with an asterisk. The majority of the studies (30/37) was judged to have described the intervention in detail. Where applicable/where addressed, most studies (13/14) were assessed to have avoided contamination through inadvertent treatment but not to have avoided co-interventions (17/23) as in many cases, subjects were taking medication during the study period (e.g. analgesics, laxatives in anorectal disorders). Most studies (34/37) reported results in terms of statistical significance, chose analysis methods appropriate for the study and the outcomes (32/35) and reported on drop-outs (26/37). All studies (37/37) were assessed to have discussed the relevance of the results to clinical practice and the majority of the studies (32/37) were judged to draw appropriate conclusions, given the study methods and results. The arbitrary sum score ranged between 5 and 13 (mean 9.2).

Table 9 shows the quality assessment of the 9 RCTs according to the PEDro scale [81], resulting in 2 studies of fair [26, 55] and 7 studies of good [16, 21, 42, 44, 45, 47, 48] quality. The mean PEDro score of these studies was 6 (range 5–8). All studies were randomized (9/9), analyzed the between-group difference (9/9), reported point estimate and variability (9/9) and had similar groups at baseline (9/9). Some studies had a concealed allocation (4/9), 4 out of 9 studies reported adequate follow-up. The majority of the studies did not have blinded participants (8/9), blinded therapists (9/9) or blinded assessors (5/9). In 7 out of 9 studies all subjects for whom outcome measures were available received the treatment or control condition as allocated or, if this was not possible, data for at least one key outcome were analyzed by intention to treat [81].

**Table 9:** Methodological quality assessment, evaluating the included randomized controlled trials (*n* = 9): Physiotherapy Evidence Database (PEDro) scale [81]

Criteria	Eligibility criteria and source	Random allocation	Concealed allocation	Baseline comparability	Blinding of subjects	Blinding of therapists	Blinding of assessors	Adequate follow-up (>85%)	Intention-to-treat analysis	Between-group statistical comparisons	Reporting of point measures and measures of variability	Total score	Quality
Chiarioni et al. 2010 [16]	Yes	Yes	Yes	Yes	No	No	Yes	Yes	Yes	Yes	Yes	8	Good
Chiarioni et al. 2006 [21]	Yes	Yes	Yes	Yes	No	No	Yes	No	No	Yes	Yes	6	Good
Ba-Bai-Ke-Re et al. 2014 [26]	Yes	Yes	No	Yes	No	No	No	No	Yes	Yes	Yes	5	Fair
Philips et al. 1992 [42]	Yes	Yes	No	Yes	No	No	Yes	Yes	Yes	Yes	Yes	7	Good
Bennink et al. 1982 [44]	Yes	Yes	No	Yes	No	No	No	Yes	Yes	Yes	Yes	6	Good
Vagedes et al. 2019 [45]	Yes	Yes	Yes	Yes	No	No	No	No	Yes	Yes	Yes	6	Good
Lúcio et al. 2014 [47]	No	Yes	No	Yes	Yes	No	Yes	No	No	Yes	Yes	6	Good
Aalaie et al. 2020 [48]	Yes	Yes	Yes	Yes	No	No	No	Yes	Yes	Yes	Yes	7	Good
Kent et al. 2015 [55]	Yes	Yes	No	Yes	No	No	No	No	Yes	Yes	Yes	5	Fair

PEDro scale: 11 items; total score: 10; eligibility criteria item does not contribute to total score; codes: yes = 1, no = 0; quality score: < 4 = poor quality, 4–5 = fair quality, 6–8 = good quality, 9–10 = excellent quality [80]

Table 2 gives an overview of the characteristics of the included studies, additionally outlining study design, comparison characteristics and sample sizes.

#### Participants

A total of 2913 patients with pelvic pain conditions and 75 healthy subjects were included in 37 studies, of whom 2489 patients were assigned to groups receiving biofeedback. The other subjects received different treatment, no intervention or standard care (Table 4).

Table 3 (and Table 2) present the patient characteristics: 5 studies investigated patients with anorectal pain syndromes [16–20], 11 studies evaluated patients with constipation [21–31], 4 studied men with nonbacterial chronic prostatitis [32, 34–36], 12 investigated females with CPP (vulvar vestibulitis syndrome/dyspareunia, pelvic floor dysfunction, dysmenorrhea, sexual dysfunction, or urethral syndrome) [37–45, 47, 48], 1 evaluated patients with low back pain [55] and 4 studied children with pelvic floor spasm [51], overactive bladder syndrome [52], dysfunctional voiding [53] or pubertal chronic prostatitis [54]. Overlapping diagnoses were common. The literature search only revealed chronic (no acute) pelvic pain conditions treated with biofeedback. The majority of the studies (24/37) stated that a secondary cause of pelvic pain had been excluded [16–20, 22–28, 30, 32, 34–36, 38, 41, 42, 48, 52–54]. One study enrolled patients with multiple sclerosis as an underlying disease [47], 6 studies [18, 20, 23, 25, 27, 30] indicated that the included subjects suffered from some kind of psychopathology (anxiety, depression, emotional trauma), 3 studies explicitly excluded patients with a psychopathologic disorder [16, 45, 48]. A total of 15 studies [18, 20, 21, 23–28, 32, 35, 38, 39, 42, 52] stated that conventional treatment including medication, changes in diet and interventions had failed prior to biofeedback.

Age ranged between 11 and 96 years in studies mainly enrolling adults. The mean age for trials involving children was 8.4 years [51–53] and 16.5 years for the study investigating adolescents [54].

#### Intervention

Table 4 presents an overview of the study intervention characteristics. 27 study protocols applied biofeedback only (together with counselling/education, pelvic floor exercises and home exercises, which are counted as part of the biofeedback intervention) [17, 18, 20–32, 34, 36, 38, 39, 42–45, 48, 51, 53, 54], others applied biofeedback as a multimodal treatment component (including psychological techniques [16, 19], electrotherapy [35, 37, 40, 41, 46, 47], medication [37], manual therapy [40] or guidelines-based care [51, 55]). Most studies evaluated outcome after the treatment, some (re)evaluated 2–3 months after the end of the treatment [16, 21, 24, 26, 35, 36, 41–43, 48], some had a long-term follow-up (6–mean 28 months) [16, 18, 21–28, 32, 38, 51].

Anorectal manometric systems and surface EMG techniques were the commonly applied anorectal physiological assessment tools in studies dealing with anorectal disorders. Male chronic pelvic pain syndromes used EMG-guided training [32, 34–36]. In urogenital phenotypes in children and adolescents, both urodynamics and perineal EMG were used. In female chronic pelvic pain syndromes, most studies used pelvic floor EMG to evaluate pelvic floor function. Three studies on patients with dysmenorrhea [43–45] aimed at increasing general relaxation by using heart rate variability training, skin temperature training and EMG of the frontalis and lower abdominal muscles.

Overall, the biofeedback training extent was largely heterogeneous, 2–30 sessions were administered, lasting between 10 and 60 min, for up to 6 months. Most designs applied biofeedback weekly, less often sessions were scheduled twice or three times a week or once every 2 weeks. Biofeedback in a home-based setting was applied daily in 3 studies on gynecological disorders [38, 39, 45]. Treating anorectal disorders, four large trials by Chiarioni et al. [16, 21, 28] and Ba-Bai-Ke-Re et al. [26] proved 5 weekly biofeedback sessions of 30min to be successful (Table 4).

Of the studies 11 reported that no biofeedback-related side effects had occurred [16–19, 21, 25, 29, 30, 35, 48, 52] and 1 study noted a transient skin irritation related to the use of a tape [55].

#### Outcome

##### Primarily evaluated outcomes: pain intensity, overall symptom improvement, quality of life

Heterogeneous assessment methods were used to evaluate primary outcome measures within a certain phenotype (Tables 5 and 7). Pain was assessed using either visual analog scale (VAS) or numeric rating scale (NRS) [16, 17, 32, 35, 37–40, 45, 55, 60] or subdomains of relevant questionnaires [29, 34–36, 47, 48, 54]. In terms of overall symptom improvement, several studies used symptom scores [26, 32, 34–36, 41, 43, 44, 47, 48, 52–54]. Apart from using standardized questionnaires, many studies reported the success rate, given as the number or percentage of patients who stated subjective pain or symptom improvement. Definitions regarding the extent of symptom improvement differed between studies (Tables 5, 6 and 7).

Quality of life was only assessed in 9 studies [26, 29, 34–36, 40, 41, 45, 54], applying questionnaires, subdomains of validated symptom scores or impact on quality of life on a VAS or NRS scale [60].

Outcome tools together with references of the respective questionnaires are outlined in Tables 5 and 7.

##### Secondarily evaluated outcomes: physiological parameters

Pelvic floor function was assessed using manometric devices, urodynamic devices as well as surface EMG techniques and digital examination. One study observed general relaxation through heart rate variability measures [45].

### Effect of biofeedback interventions on pain, overall symptoms

Table 5 presents the effect of biofeedback-assisted interventions on pain and overall symptom improvement in detail. To provide a better overview, the main conclusions drawn by the respective authors are additionally subsumed in Table 2.

Only three [16, 17, 19] out of five studies evaluating anorectal pain syndrome provided *p*-values for pain outcomes. Significant anorectal pain relief could be shown, whereby patients who finished had superior results compared to those who discharged themselves before completion of treatment [19]. A large RCT of good quality by Chiarioni et al. 2010 found biofeedback to be superior to electrogalvanic stimulation and local massage therapy both in the short and long term, whereby these differences were only significant in patients with a highly likely levator ani syndrome (tenderness of the levator ani muscle on the rectal examination) [16].

Eleven studies investigated patients with constipation: 2 RCTs of adequate sample size studied patients with dyssynergic defecation [21, 26] and found that biofeedback significantly decreased abdominal pain compared to laxatives (polyethylene glycol) [21, 26] with long-term effects and huge effect sizes significantly different from zero [21]. The same two RCTs found biofeedback superior to laxatives in terms of constipation symptom improvement with very large effect sizes [26].

Several of the remaining nine non-RCTs found pain [22–24, 27, 29] and constipation symptoms [22, 23, 25, 27, 29, 31] improved after biofeedback, at least for certain subgroups. Studies showed contradictory results regarding the question of whether biofeedback only benefited patients with PF dyssynergia or also patients with prolonged transit time. Some studies found that biofeedback improved (long term) symptoms for pelvic floor dyssynergia [22, 24, 28] but not for slow transit constipation [24, 28], others found that both phenotypes benefited equally from treatment [23, 25, 27].

With respect to the 11 studies on female chronic pelvic pain, several could improve pain [37–40, 48] or symptoms [38, 39, 41, 43, 44, 46–48], at least in the longer term. Again, several studies lacked *p*-values or measures of clinical relevance.

The 4 urogenital studies on children and adolescents and 4 studies on men with chronic prostatitis mostly found improvements in pain [32, 34–36, 51, 52, 54] and urological symptoms [32, 34–36, 52–54], with medium to huge effect sizes in Yang et al. [35].

### Effect of biofeedback interventions on quality of life

Nine studies used biofeedback to improve pelvic floor function and found a significant improvement in the quality of life postintervention in eight trials (Table 6). The findings came along with small [29, 35] to huge [26, 36] effect sizes, with 5 studies showing a significant effect for at least some outcomes [26, 29, 35, 36, 45]. A home-based heart rate variability training failed to significantly improve quality of life compared to standard care [45].

### Effect of biofeedback interventions on physiological parameters

Table 7 presents significant changes in physiological outcome assessment following biofeedback interventions. Biofeedback training could significantly improve at least some manometric values in 9 [16, 20–22, 24–26, 28, 31] out of 10 studies on anorectal dysfunction. In Heah et al. [17] manometric values did not significantly change posttreatment. Six [16, 17, 21, 22, 24, 28] out of 9 studies did not improve resting anal canal pressures. Studies on constipation and dyssynergic defecation found that paradoxical contraction on evacuation [21, 22, 24, 28, 31] and the ability to defecate a balloon [21, 28, 31] could be improved. Patients with dyssynergic defecation could improve more manometric values than patients with slow transit constipation [28]. A landmark trial on anal pain syndrome showed that patients with a tenderness of the levator ani muscle on digital palpation could improve more manometric values than patients without tenderness on the rectal examination [16].

In female chronic pelvic pain, four studies did improve EMG values of the pelvic floor or lower abdomen [38, 39, 44, 52] whereas two (mostly) failed to do so [40, 42].

In urologic phenotypes all [36, 53] or some [52, 54] urodynamic measures could be significantly improved.

## Discussion

### Quality

This systematic review included 37 quantitative studies and found tentative evidence that biofeedback-assisted training interventions can improve the primarily evaluated outcomes pain, overall symptoms, and quality of life. Results should be considered with caution due to quality issues of many of the included trials. Only 9 studies had an RCT design, out of which 7 were judged to be of good quality according to PEDro assessment. Many studies were likely underpowered and did not provide a sample size calculation.

Biofeedback is a modality to improve self-efficacy and learning based on operant conditioning [83]. Biofeedback is not used as an intervention on its own but is rather an adjunctive tool to other standard interventions (e.g. pelvic floor exercises, education, lifestyle modification [84]). At times, studies applied biofeedback together with additional physical modalities. Besides, patients were often under medication during the study period for symptom control. Therefore, the single effect of biofeedback intervention is difficult to extract. Biofeedback protocols are difficult to compare between institutions as treatment protocols, biofeedback devices and training amount varied considerably.

Most studies compare improvements within an intervention group which reduces the strength of evidence. Most authors drew their conclusions based on the statistical significance, only two papers [45, 48] reported on effect sizes and confidence intervals of pain and symptom outcomes. Ten studies [21, 26, 29, 35, 36, 40–44] provided data to calculate effect sizes and confidence intervals to evaluate the clinical relevance of the results [85]. The majority of the studies did not perform a post hoc analysis or a correction for multiple testing. Some studies used nonvalidated outcomes to evaluate pain and overall improvement.

The impossibility to fulfil certain quality requirements such as blinding of participants or the administration of placebo treatment, which are standard in pharmacological studies, is immanent to the biofeedback training method and setting.

Given these limitations, the statements that were drawn conducting this review should be understood as tentative evidence and should be considered with caution. Three RCTs of above-average quality with respect to sample size, study design, and reporting [16, 21, 45] are given special attention in the subsequent discussion.

### Efficacy of biofeedback in certain phenotypes and existing recommendations of guidelines

For anorectal disorders, such as dyssynergic defecation and levator ani syndrome, guidelines exist that state that biofeedback is the preferred treatment for chronic anal pain syndrome (level of evidence IA), [2] and is considered useful in the short-term treatment of levator ani syndrome with dyssynergic defecation (level of evidence IIB) [83]. Biofeedback is recommended for the short-term and long-term treatment of constipation with dyssynergic defecation (level of evidence IA), which is the most common defecation disorder, affecting about 40% of patients with chronic constipation [83]. Biofeedback seems to benefit patients with dyssynergic defecation above other types of constipation [24, 28, 83, 86]. In PF dyssynergia, a landmark trial by Chiarioni et al. [21] found biofeedback to be superior to laxatives (polyethylene glycol), two other RCTs [87, 88] (not considered in this review) also considered it superior to alternative treatments (diazepam), placebo, sham feedback and standard treatment [86]. The pathophysiology of levator ani syndrome seems to be similar to that of dyssynergic defecation, thus similar techniques and protocols have been used [16]. Both EMG and pressure-based biofeedback therapy protocols appear to be efficacious in restoring a normal pattern of defecation, but larger comparative trials are lacking [83]. Surface EMG probes are cheaper, more durable and usually provide a one or two-channel display [83]. Manometric systems are more expensive, have a multiple channel display and can facilitate rectoanal coordination and sensory training because they have a balloon and rectal sensor [83, 86].

In patients with vulvar vestibulitis syndrome (vulvodynia, dyspareunia), preliminary evidence has suggested that altered muscle abnormalities (as shown by altered EMG activity such as elevated resting activity, reduced muscle contraction strength, muscle instability) are present and EMG biofeedback muscle rehabilitation, therefore, is beneficial [1, 38]. According to Mariani, biofeedback should be used as a first-line treatment in moderate to severe vulvar vestibulitis (together with antidepressants and psychological counseling) [89]. Two uncontrolled studies by Glazer et al. and McKay at al. using portable EMG biofeedback devices showed promising results with this indication [38, 39]. Bergeron et al. (not considered in this review) applied the home-based Glazer protocol in an RCT design. They confirmed that EMG biofeedback as well as cognitive-behavioral therapy and vestibulectomy, could improve sexual function and reduce pain (greatest pain reduction in the vestibulectomy group [1, 90, 91]) in the short and long term.

### Pros and cons of biofeedback in pelvic pain conditions and criteria to improve treatment success

Biofeedback is a safe method, which has not shown any significant adverse effects. This might make biofeedback an attractive treatment option even in indications with a smaller success rate. As biofeedback is a labor-intensive approach [83] and quite time-consuming for both therapist and patient, it is important to preselect those patients who have a high chance of benefitting from the intervention.

The use of biofeedback to treat pelvic pain is based on the idea that these pain conditions may result from, or are associated with, pelvic floor muscle dysfunction. Digital palpation of pelvic floor muscles should be integrated into routine examination to identify myofascial pain as a primary or contributing source of pelvic pain condition [2, 8]. In anorectal pain conditions, tenderness on rectal examination has shown to be a valid criterion of treatment success [16]. Shoskes et al. identified and grouped six clinical phenotypes (urinary, psychosocial, organ-specific, infection, neurologic, tenderness of skeletal muscles) in the UPOINT classification in patients with urologic CPPS [2, 92, 93]. This classification was implemented to help direction therapy according to phenotypes, thereby improving outcomes [93]. Thus, patients with a musculoskeletal phenotype can be selected who most likely benefit from biofeedback interventions.

In patients with constipation, biofeedback therapy seems to benefit especially patients with dyssynergic defecation [21, 24, 28, 83, 86–88].

Another criterion of success might be a center’s capacities to administer a certain amount of training sessions and the patient’s willingness to complete the course of therapy as suggested by the therapist [19]. In patients with chronic constipation and dyssynergic defecation, consensus guidelines on biofeedback therapy [83] recommend 4–6 biofeedback sessions to manage dyssynergic defecation accordingly: 3 sessions [31] achieved a symptom improvement of only 45.3% compared to e.g. 80% achieved by 5 sessions in Chiarioni et al. [21], hence following existing consensus recommendations improves outcome. As biofeedback requires commitment on the patient’s part to take responsibility for their own health, the patient’s motivation and adequate encouragement to complete the course of therapy through the therapist are other important requirements for the therapeutic success [29, 36]. Cognitive impairment in the older population might lead to slower learning and the need for a higher number of treatment sessions [30]. Medical staff should be capable of demonstrating and explaining the method according to the patient’s comprehension and education levels [36]. Similarly, counteracting problems of comprehension by using appropriate explanations and psychological approaches are important in the work with children [54].

The effectiveness of pelvic floor biofeedback training also depends in part on the skills and experience of the biofeedback therapist and the particular techniques that are used to perform the training [28], which is why it is recommended to follow existing consensus guidelines [83].

As the access to biofeedback remains limited in many areas [31] and only a few centers offer biofeedback therapy, home-based self-training program is desirable and is a promising approach in anorectal and gynecologic (vulvar vestibulitis syndrome) disorders [38, 39, 83], at least to continue training after initial training at a center.

With somatoform disorders and related syndromes, the etiology is still not fully understood but evidence supports an interaction of physiological, psychological and interpersonal factors [1]. Therefore, a multimodal treatment strategy can be promoted, using biofeedback, relaxation training and stress management to address physiological and emotional arousal as well as cognitive techniques, psychoeducation and attention training to alter cognitive-perceptual factors, a modification of illness behavior and graded activity [1]. Multidisciplinary management, which is a common approach to many chronic conditions, is still not commonly available in gynecology because of cost factors and limited availability of interested specialists [7]. Yet multidisciplinary, multimodal and phenotype-oriented approaches have been increasingly proposed to deal with gynecologic phenotypes, such as provoked vestibulodynia and myofascial pain as well as with other chronic pelvic pain conditions such as bladder or prostate pain syndrome [2, 8, 90, 93].

Hence, biofeedback is not a complementary or alternative but an additive method for both diagnostic and therapeutic purposes. It should be used in addition to standard care, based on a state of the art concept, if the physician in charge gives the indication.

### Limitations of the present review

The authors decided to include any quantitative study type of primary research to present a comprehensive overview of the current literature. This reduces the methodological quality of the trials and thereby the significance of the results.

The search term “pelvic pain” is wide-ranging, yet there are many terms used in literature to describe pain syndromes which are perceived in a certain organ [94] and specific pathologies that cause pelvic pain. Therefore, our pragmatic and generalized search strategy carries the risk of missing relevant articles. Studies evaluating biofeedback on constipated patients were included, yet constipation was not the primary focus of this paper as the pain component is not paramount; however, this phenotype has been researched in depth, and our search term did not reveal all relevant studies available in the literature. As with the phenotype of dyspareunia, the reader is referred to the respective relevant literature [14, 83, 86, 90].

Due to language restrictions, studies that would have otherwise fulfilled the inclusion criteria could not be included.

## Conclusion

Several landmark studies demonstrated the efficacy of biofeedback for anorectal disorders. For other phenotypes of chronic pelvic pain, there is tentative evidence that biofeedback-assisted training interventions can improve the outcomes on pain, overall symptoms, and quality of life. Clinical improvements came along with improvements in certain physiological parameters in several studies. Many trials were characterized by methodological limitations, such as a very small sample size, nonvalidated outcomes and a lack of control group. The preliminary positive findings should be investigated further in robust and well-designed randomized controlled trials. Certain factors have been identified that might be relevant for improving biofeedback treatment success.

### Implications for future research

Future studies should aim to:

- conduct a systematic literature review using MeSH terms that more thoroughly evaluate the effect of biofeedback therapy in a certain phenotype (e.g. anorectal disorders, urological chronic pelvic pain syndrome, bladder pain syndrome, gynecologic pelvic pain conditions);
- list the term “pelvic pain” in the keywords of studies on certain pelvic pain subtypes so that these trials are detected by a literature search on the umbrella term (as chronic pelvic pain comprises many phenotypes);
- improve the quality of future studies, e.g. by choosing an RCT study design that is based on a sample size calculation, performing a post hoc analysis or a correction for multiple testing;
- report on the effect size and an estimate of their precision such as the confidence interval to describe the clinical relevance of results;
- conduct future trials with more homogeneous outcome assessment (to allow future meta-analysis). Ideally, validated questionnaires or pain scales should be used to measure outcome. For stating success rates, an international consensus on the graduation of these rating scales would be beneficial to standardize outcome and improve comparability between study results;
- continue to evaluate the optimum type and extent of biofeedback interventions for the certain phenotypes;
- evaluate changes in quality of life and psychological parameters, such as anxiety and depression (as psychological disorders are common comorbidities in patients with chronic pain conditions).
